# Revealing neuronal function through microelectrode array recordings

**DOI:** 10.3389/fnins.2014.00423

**Published:** 2015-01-06

**Authors:** Marie Engelene J. Obien, Kosmas Deligkaris, Torsten Bullmann, Douglas J. Bakkum, Urs Frey

**Affiliations:** ^1^RIKEN Quantitative Biology Center, RIKENKobe, Japan; ^2^Graduate School of Frontier Biosciences, Osaka UniversityOsaka, Japan; ^3^Department of Biosystems Science and Engineering, ETH ZurichBasel, Switzerland

**Keywords:** microelectrode array, neuronal function, extracellular recording, stimulation, CMOS, multielectrode array, neuron-electrode interface, multi-scale modeling

## Abstract

Microelectrode arrays and microprobes have been widely utilized to measure neuronal activity, both *in vitro* and *in vivo*. The key advantage is the capability to record and stimulate neurons at multiple sites simultaneously. However, unlike the single-cell or single-channel resolution of intracellular recording, microelectrodes detect signals from all possible sources around every sensor. Here, we review the current understanding of microelectrode signals and the techniques for analyzing them. We introduce the ongoing advancements in microelectrode technology, with focus on achieving higher resolution and quality of recordings by means of monolithic integration with on-chip circuitry. We show how recent advanced microelectrode array measurement methods facilitate the understanding of single neurons as well as network function.

## Introduction

Studying the function and connectivity of neurons in the brain involves coordinated, interdisciplinary efforts among scientists from various fields. Through the years, advancements in genetic markers, immunostaining, optical and electro-optical methods, electrophysiology, and computational tools have been made to identify neuronal types, explain their molecular machinery, untangle their wiring, decipher principles of neural coding, and to attribute functional roles to specific regions of the brain. The brain is a complex system and its activity spans over multiple temporal and spatial scales that require a comprehensive set of technologies to address these scales. Innovations in experimental methods to observe and perturb brain activity and in computational tools to analyze recorded data are needed to master the brain's complexity and advance our understanding of its function. Systems biology has allowed to bridge between molecular dynamics and whole cell simulations using multi-scale modeling. Applying similar approaches to brain activity will allow us to gain a more encompassing understanding of it. However, quantitative data at all these spatial and temporal scales are a prerequisite.

**Figure 1:**
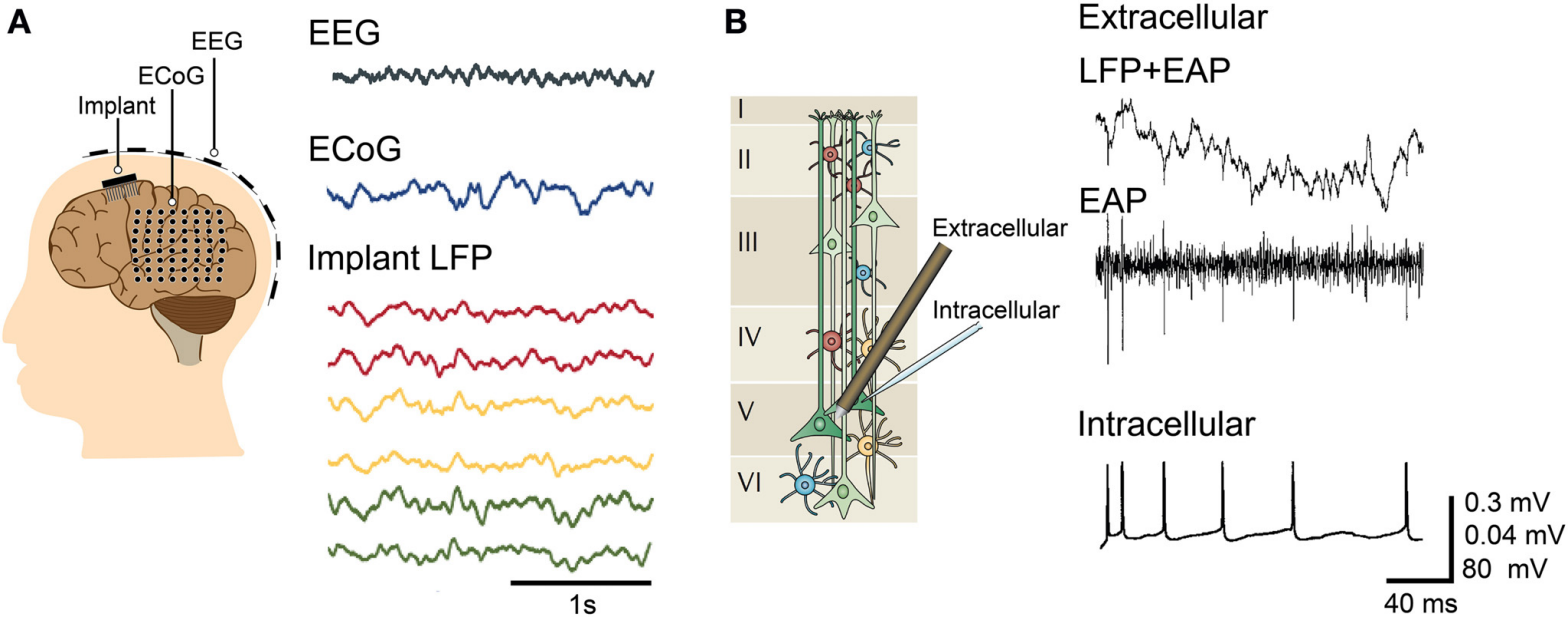
**Typical electrophysiological methods**. **(A)** Macroscopic recording via electroencephalography (EEG) and mesoscopic recording through electrocorticography (ECoG) and implantable electrodes, with the corresponding representative waveforms recorded in a patient with drug-resistant epilepsy. The measured signal amplitudes are larger for ECoG and implanted electrodes (local field potential or LFP recording) compared to EEG. The waveforms for EEG, ECoG, and implant are modified with permission from Buzsáki et al. (2012). **(B)** Mesoscopic and microscopic recording using a tetrode (extracellular) and a glass micropipette (intracellular), respectively. The fast EAP extracted from the raw tetrode recordings correlate with the intracellular APs recorded from a pyramidal cell. (Left) Illustration of cells across cortical layers modified with permission from Buzsáki et al. (2012). (Right) Signals for simultaneous extracellular and intracellular recordings modified with permission from Henze et al. (2000).

Electrophysiology has been the preferred means of analyzing brain activity due to the ability to capture a wide range of neural phenomena, from the spiking activity of individual neurons to the slower network oscillations of small populations (Llinás, 1988; Contreras, 2004; Assad et al., 2014). The electrical nature of neuronal activity makes it possible to detect signals on electrodes at a distance from the source, but not without caveats. It is necessary to determine the recording capabilities and limits of the device used and to understand how the neuronal signal is transduced into a recorded digital form. Typical electrophysiological methods are shown in Figure 1 and further described below.

At the microscale, patch-clamp can be used to measure currents of single ion channels. The function of single neurons is often explored by direct measurements of the intracellular voltage, using patch-clamp or a sharp microelectrode. It is a powerful but tedious method and often its use is limited to a few neurons per experiment (Wood et al., 2004). Planar patch-clamp systems allow rapid *in vitro* patch-clamping, mostly used for high-throughput ion channel screening of dissociated cells (Dunlop et al., 2008). Automated patch-clamp allows for fast *in vivo* intracellular recording and it is feasible to extend the method to measure several neurons simultaneously (Kodandaramaiah et al., 2012). The bulkiness of current micromanipulators and patch-clamp systems together with the necessity for accurate and precise control have limited simultaneous patch-clamp recordings to a few—maximum of four and twelve for *in vivo* (Kodandaramaiah et al., 2014) and *in vitro* (Perin et al., 2011), respectively.

At the macroscale, indirect measurement of large areas of the brain's activity is achieved via functional magnetic resonance imaging (fMRI), positron emission tomography (PET), and electroencephalography (EEG). These methods can be used to resolve functional connectivity among brain regions. For example, EEG detects spontaneous or evoked electrical activity from the scalp with low spatial resolution (cm range).

In this review, we focus on electrophysiology at the mesoscale—extracellular recordings via metal electrodes, open-gate field-effect transistors (OGFETs) or oxide-semiconductor FET (OSFET) integrated into large arrays, so-called microelectrode arrays (MEAs). This method enables simultaneous and long-term recordings of local field potentials (LFPs) and extracellular action potentials (EAPs) from a population of neurons at millisecond time scale. It also allows perturbing neuronal activity using electrical stimulation. As data obtained from *in vivo* and *in vitro* experiments are often very similar, the MEA technology, concepts, and applications we include here apply to both and will be helpful for scientists and engineers from either field. In particular, we explain the interface between the neuron and the electrode in order to understand how to interpret the recordings. We highlight trends in the development of complementary metal-oxide-semiconductor (CMOS) based high-density MEAs (HDMEAs). The advantages of HDMEAs include the capability to map neuronal activity at sub-cellular resolution, localize single cells, and to constrain full-compartmental neuron models.

The outline is as follows. Chapter 2 gives an overview of the MEA technologies, including the comparison between *in vivo* and *in vitro* MEA devices from a technical aspect. Chapter 3 describes the current understanding on microelectrode recordings and introduces the different factors that shape the recorded signals. Chapter 4 discusses how to process MEA signals and reviews recent works on using MEAs for neuroscience studies. We then conclude in Chapter 5 with perspectives on advanced measurements and applications of MEAs for studying neuronal function.

## MEA technology

This chapter reviews the technology involved in MEA development.

### Device types and terminology

Over the years, a wide repertoire of terms has been used to refer to and distinguish between all the different forms of MEAs, e.g., emphasizing the type of transducers used (multi-transistor array, microelectrode array, multielectrode array, micro-nail array, capacitive-coupled array, 3D MEA), the type of substrate (active array, passive array, silicon array, CMOS array), the shape of the device (needle-type probe, polytrode, neuro dish), the channel count (multichannel array), the electrode density (HDMEA) or the application (implantable array, *in vivo* MEA, *in vitro* MEA) and more. We would therefore like to briefly explain the terminology used in the context of this review. We generalize the term microelectrodes and MEA to cover both substrate-integrated planar MEAs and implantable neural probes. We also include capacitive-coupled devices, such as multi-transistor arrays in the definition of MEAs. We then distinguish between implantable, *in vivo* MEAs, such as polytrodes and neural probes, and *in vitro* MEAs that generally include a cell culture dish or some other sort of medium chamber. Further, we classify the different array architectures, as will be explained in Section Advances in MEA and Probe Devices (**Figure 3**). Briefly, we distinguish between “fixed wiring” arrays, meaning that each transducer in the array has a *direct wire* to the outside of the array and “multiplexed arrays,” in which some sort of switching mechanism is employed *within* the array. We use the term “array” to refer to the actual area that encompasses the transducer elements only and we use device or MEA to refer to the entire device. With system, we refer to the MEA and all required components to operate it, such as the data acquisition hardware and software. We use the terms “active” and “passive” to distinguish between devices with active circuit elements, such as transistors, and devices without such elements.

### Electrodes and transducers

There are various techniques for fabricating microelectrodes, which are reviewed by Li et al. (2003), Park and Shuler (2003), Huang et al. (2009). Choosing the materials for the insulator, conductor, microelectrode, and substrate is crucial, in particular with respect to biocompatibility. All materials in the MEA that will be near to or in contact with cells and tissue need to be tested for toxicity in prolonged periods of time (Hassler et al., 2011). It is also important to consider the biological experiments for which the microelectrodes will be used, whether *in vivo* or *in vitro*, culture or acute preparation. Moreover, deciding the type of MEA to use is highly dependent on the type of recorded signals needed, whether EAPs and/or LFPs or intracellular action potentials (IAPs), single cell resolution or not. If the MEA is to be used for stimulation, the charge capacity of electrodes is an important aspect. The electrode needs to be able to mediate reactions at the electrode-electrolyte interface to allow electron flow in the electrode to transition into ion flow in the electrolyte toward stimulating nearby cells (Cogan, 2008).

Generally, an important goal of electrode fabrication is to achieve low impedance. Low electrode impedance results in higher signal-to-noise ratio (SNR), with the usual target SNR of 5:1 or higher. Uniformity of the electrode impedance across an array of electrodes may also be important to obtain consistent data.

Typically, electrodes are made with metallic conductors such as gold (Au), titanium nitride (TiN), platinum (Pt), stainless steel, aluminum (Al), and alloys like iridium oxide (IrOx). Since the electrodes used in MEAs are on the micrometer scale, it is a challenge to achieve low electrode impedance with plain conductors only. Increasing the effective surface area of electrodes can be achieved by modification with porous conductive materials such as Pt-black, Au nanostructures, carbon nanotubes (CNTs), and conductive polymers like poly(3,4-ethylenedioxythiophene) (PEDOT). Emerging materials aside from PEDOT and CNTs include doped diamond and graphene. By modifying the surface, the electrode impedance can be decreased drastically and neuronal recording can be improved (Cui et al., 2001; Franks et al., 2005; Ludwig et al., 2006; Keefer et al., 2008; Viswam et al., 2014). Nam and Wheeler (2011), Kim et al. (2014) for a review of electrode materials and surface modification.

Non-metallic electrodes have been mostly used in conjunction with field-effect transistor (FET) based transducers (Bergveld, 1970; Fromherz et al., 1991). An OGFET can, e.g., be obtained if the fabrication process of a FET is stopped before depositing the gate material (Jenkner et al., 2004). Easier to fabricate is the so-called extended-gate FET (EGFET), in which the FET is fabricated without modification from a standard CMOS process. Metal and via interconnections are used to extend the gate to the surface of the chip, where an insulated electrode implements the “extended gate.” Such insulation ensures that no faradaic currents occur. However, as Hierlemann et al., pointed out, devices with metal electrodes also usually connect to a FET directly (Imfeld et al., 2008) or through a filter capacitor (Heer et al., 2006), resulting in a largely capacitive recording situation (Hierlemann et al., 2011). OGFET, EGFET, and devices that directly connect the electrode to the first FET usually need to include some measures to properly bias the gate or some calibration mechanism, which may cause transient currents to flow at the electrode. Whereas for devices with a capacitively coupled front-end stage, controlling the electrode input node is generally not needed. Devices with a FET-based transducer, but using a metalized gate exposed to the liquid, have also been developed (Jobling et al., 1981).

Recently, ultra-small electrodes are being developed to record intracellular activity, including subthreshold signals, as reviewed in Spira and Hai (2013). This is achieved by 3D structured electrodes such as silicon nanowires (Robinson et al., 2013) and Au mushrooms (Hai et al., 2009) penetrating the cell membrane. Electroporation was shown to facilitate measurement of intracellular activity (Koester et al., 2010; Hai and Spira, 2012).

### Advances in MEA and probe devices

Since the single extracellular microelectrodes used in the middle of the last century (Weale, 1951; Gesteland et al., 1959), development quickly proceeded to MEAs with multiple transducers for the purpose of increasing the number of neurons observed (Thomas et al., 1972; Gross et al., 1977; Pine, 1980; Csicsvari et al., 2003) to increase reliability of spike sorting (Gray et al., 1995; Harris et al., 2000) and to allow for source localization (Blanche et al., 2005; Chelaru and Jog, 2005; Frey et al., 2009b; Somogyvári et al., 2012; Delgado Ruz and Schultz, 2014). The advances in lithographic techniques, fueled by the semiconductor industry, allowed a gradual increase in performance and reliability of such multichannel devices. Passive transducer devices based on electrodes embedded in glass or silicon substrates with fixed wiring to amplifiers for *in vitro* and also *in vivo* applications became commercially available in the late 90 s and early years of this century. Already early on, silicon-based biosensors for interfacing cells with microelectronics were developed (Bergveld, 1970; Parce et al., 1989). Active devices, employing FETs were fabricated and 2D arrays demonstrated (Besl and Fromherz, 2002). Devices using CMOS technology were fabricated in academic facilities (DeBusschere and Kovacs, 2001) and industrial foundries, usually in conjunction with additional processing steps for biocompatibility reasons (Berdondini et al., 2002; Eversmann et al., 2003b; Franks et al., 2003).

The key advantage of integrating active electronic components on the same substrate as the actual electrodes is the possibility of a much higher electrode number and density. Due to the possibility of using active switches to time multiplex signals, integrated circuits make it feasible to transfer data from such high channel counts off chip and to overcome the connectivity limitation of passive devices. Additionally, such co-integration allows amplifying the signals with optimal quality, due to minimal parasitic capacitances and resistances (Hierlemann et al., 2011). The monolithic co-integration also allows including additional functionality, e.g., on-chip spike detection, closed-loop capabilities, electrical stimulation, electronic chip identification, device calibration, and other type of sensing modalities, such as temperature, pH or optical sensing (Baumann et al., 1999; Tokuda et al., 2006; Johnson et al., 2013b).

**Figure 2:**
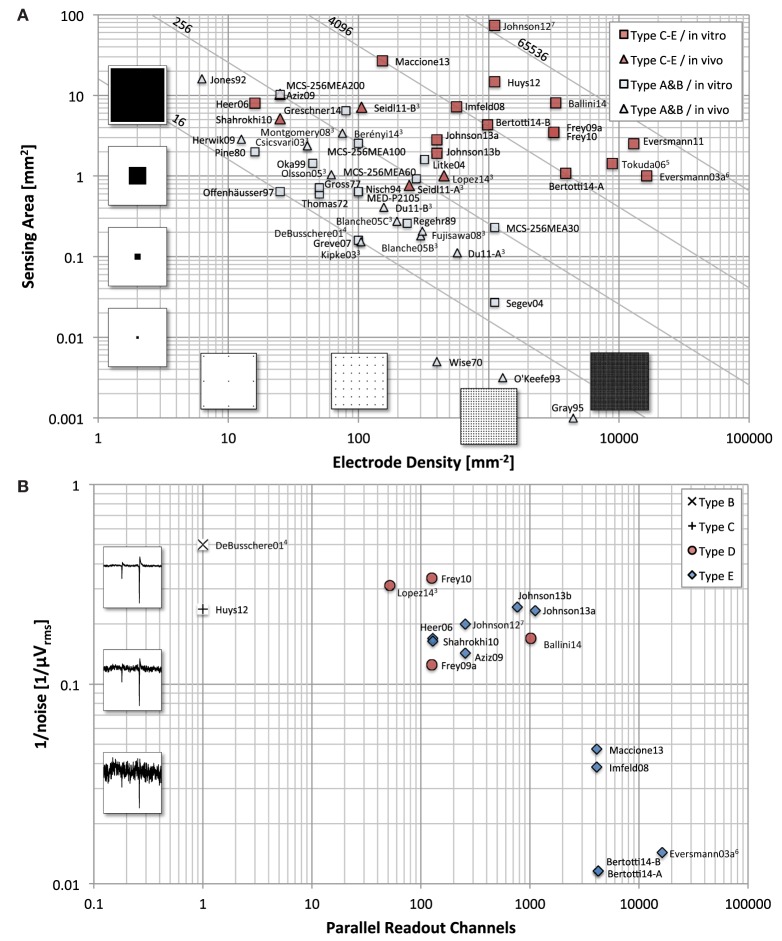
**Device comparison**. MEA comparison with respect to **(A)** electrode density and total sensing area, and **(B)** parallel recording channel count and noise level. **(A)** For devices with a regular sensor pitch, such as most *in vitro* MEA devices, the total area is calculated as number of electrodes times the pixel area. For all devices, the number of electrode times the inverse of the electrode density matches the total area. The light gray lines illustrate the number of electrodes. **(B)** The noise values shown are approximated RMS values stated in the respective citations. The conditions under which these measurements were taken usually differ significantly (such as noise bandwidth, in- or exclusion of electrode noise, inclusion of ADC quantization noise, etc.). Therefore, this graph only serves as a rough comparison. The waveforms to illustrate the noise levels are simulated and have a spectrum typical for MEA recordings. The simulated spikes are typical spikes for acute brain slice measurements recorded with microelectrodes. The recorded amplitudes may vary significantly depending on preparation and sensor characteristics. See Footnotes:^3^,^4^,^5^,^6^,^7^.

**Figure 3:**
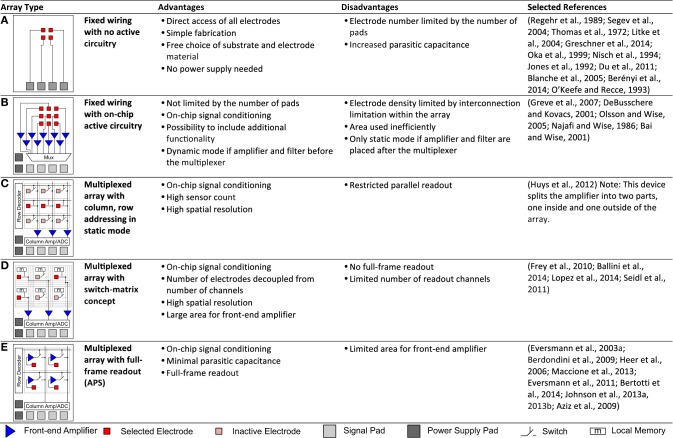
**Array architectures**. This table summarizes and classifies the different architectures that are typically used for MEAs. Advantages, disadvantages are stated and representative selected references given. **(A,B)** Fixed wiring. **(A)** Electrodes are directly connected to signal pads with no active circuitry. **(B)** Electrodes are directly connected to on-chip active circuitry for signal conditioning. **(C–E)** Multiplexed arrays. **(C)** Signals are multiplexed to the signal pads via column, row addressing in static mode. **(D)** More flexible addressing is achieved by adding more routing resources within the array in the switch-matrix mode. **(E)** All electrodes can be sampled at fast speeds in full-frame readout implemented in active pixel sensor (APS) MEAs.

Figure 2A compares a variety of historical and current devices, to illustrate the evolution of MEAs with respect to overall sensing area and electrode densities. The electrode count is shown with solid lines. The devices are categorized into fixed wiring (Type A&B in Figure 3) and multiplexed arrays (Types C–E in Figure 3). Fixed-wiring arrays include devices without any on-chip circuitry (Alpha MED Science Co., Ltd.^1^; Multi Channel Systems GmbH^2^; Thomas et al., 1972; Gross et al., 1977; Pine, 1980; Regehr et al., 1989; Nisch et al., 1994; Oka et al., 1999; Litke et al., 2004; Segev et al., 2004; Greschner et al., 2014), but also MEAs with on-chip circuitry limited to the surrounding of the array (Greve et al., 2007) and arrays that include FETs (Offenhäusser et al., 1997) and source follower devices directly wired to circuitry outside the array (DeBusschere and Kovacs, 2001). Multiplexed arrays employ some sort of multiplexing within the actual array (Eversmann et al., 2003a, 2011; Heer et al., 2006; Tokuda et al., 2006; Aziz et al., 2009; Berdondini et al., 2005, 2009; Frey et al., 2010; Huys et al., 2012; Johnson et al., 2012, 2013a,b; Maccione et al., 2013; Ballini et al., 2014; Bertotti et al., 2014).

For *in vivo* MEAs, the connectivity limitation is even more severe, as connections cannot be wired out on all four sides of the array, but only on one of the narrow sides. Figure 2A includes some examples of such devices using fixed wiring (Wise et al., 1970; Najafi and Wise, 1986; Jones et al., 1992; O'Keefe and Recce, 1993; Gray et al., 1995; Bai and Wise, 2001; Csicsvari et al., 2003; Kipke et al., 2003; Blanche et al., 2005; Olsson and Wise, 2005; Fujisawa et al., 2008; Montgomery et al., 2008; Herwik et al., 2009; Du et al., 2011; Berényi et al., 2014) and three recent *in vivo* MEAs with multiplexing on the shaft itself (Shahrokhi et al., 2010; Seidl et al., 2011; Lopez et al., 2014). For detailed reviews of *in vivo* MEAs (see Wise et al., 2008, 2004; Ruther et al., 2010).

Figure 2B, on the other hand, focuses only on CMOS-based devices and illustrates the tradeoff between the number of parallel (or quasi parallel) readout channels and the input referred noise of the amplification chain. It illustrates the fundamental fact that a low-noise front-end amplifier requires both area and power. Limiting either will inherently increase the noise levels. The power budget for the entire device, including all circuitry within the array and surrounding it, is limited by the amount of produced heat that one can tolerate. For the area constraints, one has to separately consider the area within the array and surrounding it. Within the array, the electrode density dictates the available area per pixel. Outside the array, the area is limited mostly by the fabrication cost. As a trivial approach to decouple the area requirement from the noise specifications, one can simply place the amplifiers outside the array and directly wire one electrode to one amplifier (Figure 3B). However, this approach still does not allow achieving both a high density and a large electrode count at the same time. Figure 3 lists these fixed-wiring approaches and typical array architectures using multiplexing within the array to overcome this limitation.

Active switching can be integrated into the array, allowing to time multiplex the signals from many electrodes to a few wires that carry the signals out of the array. We now consider two types of time multiplexing, static (Figures 3C,D) and dynamic (Figure 3E) operation (Imfeld et al., 2008). In dynamic mode, each pixel (or electrode) is sampled once within each frame, with typical frame-rates of 2–10 kHz for CMOS-based MEAs (Eversmann et al., 2003a; Johnson et al., 2013b) and some devices allowing as high as 77 kHz (Bertotti et al., 2014). This mode is similar to image sensors used in cameras. Typically, rectangular sub-arrays can be chosen as regions of interest and sampled at faster rates. From a circuit perspective, the challenge in designing full-frame readout MEAs lies in the fact that the multiplexing within the array requires the front-end amplifier to be located within the pixel itself, as the electrode alone exhibits a high impedance and therefore cannot drive the multiplexed readout lines at sufficient speed. Inherently, the available area within the pixels is limited in high-density arrays, making it difficult to build very low noise amplifiers. In addition, the electrodes themselves and the activity within the culture medium show wide band noise (see Section Noise and SNR), thus requiring a low-pass filter within the pixel to prevent noise from being aliased into the signal band due to the sampling. Generally full-frame readout arrays have a high channel count, and therefore the power budget per channel is very limited.

Alternative approaches to circumvent this issue and to allow for devices in which the circuit itself is not the limiting factor with respect to noise performance have been demonstrated. Arrays operating in static mode (Figures 3C,D) have only switches and no amplifiers as active devices within the array. The switches are used to wire electrodes to front-end amplifiers placed outside of the array, where sufficient area for the implementation of low-noise amplifiers is available. This also decouples the number of electrodes from the number of readout channels, which allows budgeting of the available power in more flexible ways. Devices that employ a simple column and row based static addressing are limited in the flexibility of choosing electrodes for parallel readout. A switch-matrix implementation, which consists of a large set of routing wires, routing switches, and local memory, such as SRAM cells within the array, allows the use of complex routing paths to rewire a subset of electrodes to the available readout and stimulation channels in a flexible manner. Often, such an approach is sufficient to observe biological phenomena of interest, as typically not all electrodes exhibit activity. However, experimental protocols tend to get more complex, as one needs to select the “right” electrodes during the experiment. One of the protocols commonly used for such devices is to first scan the entire array in static mode, i.e., record from each rectangular sub block for, e.g., a few minutes, run some online or quasi online data processing on the recorded data, and select a more refined subset based on the recorded activity and the scientific objective of the experiment.

Apart from the array, CMOS devices also require the design of neuronal amplifiers and some sort of data transmitter, either of the amplified analog signals or, more typically, of the already digitized data. Generally, a neural amplifier needs to have high input impedance, which is significantly higher than the electrode impedance, to ensure signal integrity. The amplifier should be of low power to prevent substrate heating that could damage cells or tissue. For *in vitro* MEA devices, a variety of target applications have to be considered. Therefore, gain and dynamic range requirements can be quite demanding and should be adjustable, such as to cover applications with maximal amplitudes of a few hundred microvolts in acute slice preparations and, on the other hand, up to 10 mV in measurements from cardiomyocytes. The same also holds true for the flexibility in the recording bandwidth. Some applications may require lower frequency signals only, some only spikes in the EAP band, some both bands with different gain requirements at the same time. The circuits need to implement some sort of high-pass filter to block the large *1/f* noise of the electrode-liquid interface typically observed. MEA systems can also include stimulation circuitry, covered in the next section, and analog-to-digital conversion (ADC). They need to include an interface to transmit the data and receive commands for controlling the system's operation. The requirements are different for implantable devices, where usually the target application is much more defined, but also the power, reliability, and safety requirements are more stringent. These systems often implement spike detection or classification and wireless transmission in the system, either as a monolithic implementation or hybrid approach using multiple ICs. They may also be powered wirelessly. On the other hand, *in vitro* MEA systems do not require wireless power or data transmission, as they can generally be directly wired to the data-receiving device. In this case, often common interface standards are employed, such as USB (Multi Channel Systems GmbH^2^), Ethernet (Frey et al., 2010), National Instrument's DAQ card (Alpha MED Science Co., Ltd.^1^), CameraLink (Imfeld et al., 2008), or others. Most of these systems support online storage of the full raw data to hard disks, sometimes including some form of lossless data compression (Sedivy et al., 2007).

Many of the circuit requirements can be traded against each other, e.g., one can easily lower the noise by increasing the area or power consumption. The key challenge therefore is to set the target specifications for the given application accurately and optimize the systems for it, without overdesigning specific requirements. Further considerations with respect to noise are given in Section Noise and SNR. Reviews focusing on circuit related issues can be found here: (Wise et al., 2004, 2008; Harrison, 2008; Jochum et al., 2009; Gosselin, 2011).

### Stimulation

MEAs allow passive observation, and also active influence and control of neuronal activity. Metal electrodes can deliver electrical stimuli directly using the microelectrodes, whereas for OGFET-based devices, typically an extra capacitive stimulation spot is used to deliver stimuli (Stett et al., 1997). In addition, monolithic CMOS integration of MEAs opens up the possibility to include electrical stimulation circuitry directly on-chip, in turn allowing a high degree of flexibility in generating spatiotemporal patterns of stimulation, higher spatial resolution for stimulation and direct on-chip stimulation artifact blanking or suppression.

Already the very first electrophysiological experiments with frogs by Galvani (1791) involved electrical stimulations using metal wires connected to various sources, e.g., Leyden jars, Franklin's magic squares, and even atmospheric electricity during lightning. *In vivo*, electrical stimulation is commonly used to stimulate nerves for transmitting sensory information to the brain, such as for cochlear implants (Wilson and Dorman, 2008) and retinal implants (Ahuja et al., 2011; Zrenner et al., 2011); to control, e.g., limbs for neurorehabilitation after nervous system injury; and to treat disorders, e.g., Parkinson's disease by deep brain stimulation using brain pacemakers (Montgomery and Gale, 2008). In such applications, the physical distance between the stimulation electrode and target nerves can be rather large, requiring the delivery of high amplitude stimuli.

Lilly et al. (1955) established charged balanced methods using biphasic brief pulses to limit the damage to the tissue and the degradation of the electrodes themselves. Merrill et al. reviewed electrical stimulation using electrodes, listing various materials (Merrill et al., 2005). For *in vitro* MEAs, effective stimulation protocols were characterized by Wagenaar et al. (2004). The authors studied different stimulation parameters (pulse width, amplitude, pulse shape) that evoke neuronal activity.

**Figure 4:**
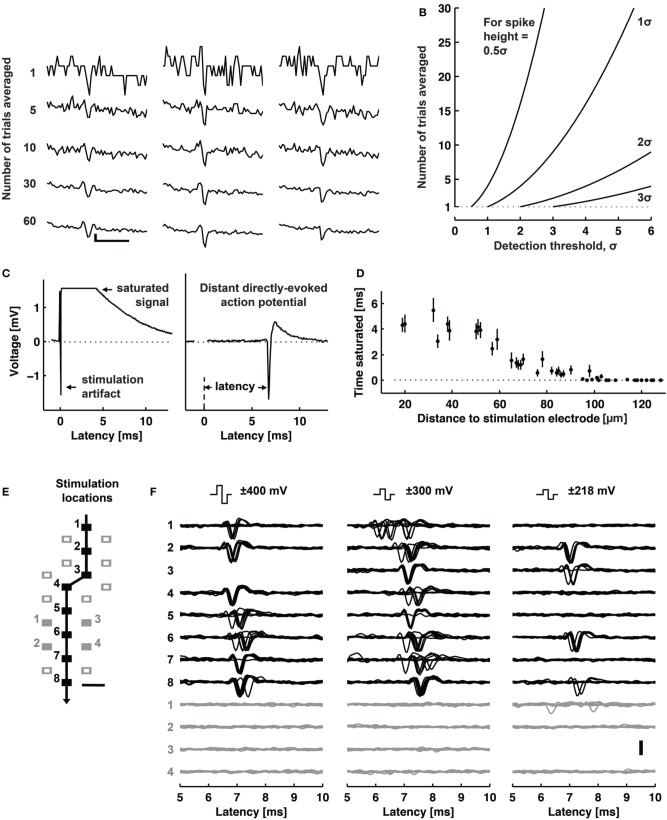
**Stimulation capability of high-resolution CMOS-based MEA**. **(A)** Examples of evoked spikes detected at three sites (columns) along the same axon. The top row shows individual raw traces, and the other rows show traces averaged as indicated. Scale bars, 1 ms horizontal, 10 μV vertical. **(B)** The amount of averaging necessary to detect a spike with a given height (0.5–3 σ) with respect to the detection threshold. **(C)** Left: A raw voltage trace recorded at an electrode neighboring a stimulation electrode saturated for about 4 ms (flat line). Right: A raw voltage trace recorded at an electrode located 1.46 mm away from a stimulation electrode did not saturate. **(D)** The duration of a saturated signal occurring after stimuli is plotted vs. distance from the stimulation electrode (mean ± s.e.m.; *N* = 18 stimulation electrodes from five CMOS-based MEAs). Stimuli consisted of biphasic voltage pulses between 100 and 200 ms duration per phase and between ± 400 and 800 mV amplitude. **(E)** Locations of stimulation electrodes that directly evoked (black boxes) or did not evoke (empty or filled gray boxes) APs detected at a soma located ~890 μm away. The line arrow indicates the orthodromic propagation direction. Scale bar, 20 μm. **(F)** Voltage traces of somatic APs elicited by biphasic voltage stimuli. Traces in response to eight stimuli are overlaid for each of three stimulation magnitudes (indicated at the top), plotted for all effective (black) and four ineffective stimulation sites (gray at the bottom). Stimulation electrode locations are represented as numbered boxes in **(E)**. Scale bar, 200 μV. All panels and description adapted with permission from Bakkum et al. (2013).

One application of electrical stimulation is the use of it as a “trigger,” so-called stimulus-triggered averaging (Cheney and Fetz, 1985). Electrical stimulation allows delivering trigger pulses of high temporal resolution in the order of a few microseconds, depending on the stimulation buffer used and the capacitive load of the electrode. Stimulation can evoke responses with small temporal jitter, e.g., Bakkum et al. observed a jitter of 160 μs using passive MEAs (Bakkum et al., 2008). Bakkum et al. used trigger signals to study the velocity of action potential (AP) propagation in axons of cultured neurons (Bakkum et al., 2013). Figure 4A shows how such stimulus-triggered averages revealed small axonal spikes of different shapes, such as bi- and tri-phasic types. Figure 4B illustrates the reduction in uncorrelated noise with increasing number of averaged repetitions. One potential issue with delivering electrical stimulation to neuronal cells and tissue is the occurrence of artifacts in recording channels, due to the fact that stimulation pulses are typically three to four orders of magnitude larger than the recorded signals. This coupling between stimulation and recording is difficult to prevent, and artifacts are picked up both within the wiring of the array and circuits, but also through the medium of the cell culture or tissue. However, as long as the coupling is purely capacitive, artifacts usually only prevent recording during the stimulation period itself. If the amplitude of an artifact is large, which can occur when a recording electrode is near the stimulation electrode, the artifact may saturate the amplification circuits of the recording electrode. This saturation will prevent recording for an extended period of time after the stimulation ended. Figure 4C shows an example of such a saturated signal from an electrode located 18 μm (center-center) away from the stimulation electrode and a signal without saturation from an electrode located about a 1 mm away. Figure 4D shows the relationship between the distance from stimulation to recording electrode and the duration of saturation for a 11,011-electrode MEA (Frey et al., 2010), without employing any artifact suppression measures. As long as the amplifiers do not fully saturate, it is possible to suppress such artifacts in software by subtracting the estimated artifact (based on templates, filters or local curve fitting) from the data (Hashimoto et al., 2002; Wagenaar and Potter, 2002). To also allow recording from electrodes on which saturation would occur, counter measures in hardware have to be employed. One solution is to use a “reset” switch that can bring back the saturated amplifier into normal operation quickly, by resetting the high-pass filter of the front-end amplifier (Heer et al., 2006; Frey et al., 2010). To suppress artifacts even on the stimulation electrode itself, more sophisticated methods are used. Jimbo et al. proposed a method to decouple the recording amplifiers during stimulation, sample the electrode potential during recording and add the stimulation pulse to the stored electrode potential (Jimbo et al., 2003). This scheme has also been implemented on dedicated ASICs to be used in conjunction with MEA devices (Brown et al., 2008; Hottowy et al., 2012; Tateno and Nishikawa, 2014). Figures 4E,F show stimuli activated neuronal responses with high spatiotemporal precision. In a study to track axonal APs (Bakkum et al., 2013) several ten thousands of stimuli were required, which was possible without damaging the electrodes or cells. In this case, voltage-mode stimulation was used, although the stimulation hardware supported both current- and voltage-mode (Livi et al., 2010).

Closed-loop experiments, in which neural activity triggers electrical stimulation, employing on-chip stimulation circuitry have been presented by Hafizovic et al. (2007) and Müller et al. (2013). In both cases, the spike detection is performed off-chip on dedicated FPGA hardware. The actual decision to stimulate and the selection of the stimulation waveform patterns is performed on a personal computer in Hafizovic et al. (2007), whereas in Müller et al. (2013) an event engine performing this task is implemented directly on the FPGA platform, making the latency until stimulation shorter and, importantly, reducing its temporal jitter.

CMOS-based devices exclusively devoted to stimulation at high spatio-temporal resolution of close to 7000 electrode per square millimeter and with variable voltage mode pulses have been developed as well (Lei et al., 2008, 2011). Circuit considerations for CMOS-based devices for clinical *in vivo* application are reviewed (e.g., Ortmanns et al., 2008; Ohta et al., 2009).

### Applications of in vitro CMOS-based MEAs

**Figure 5:**
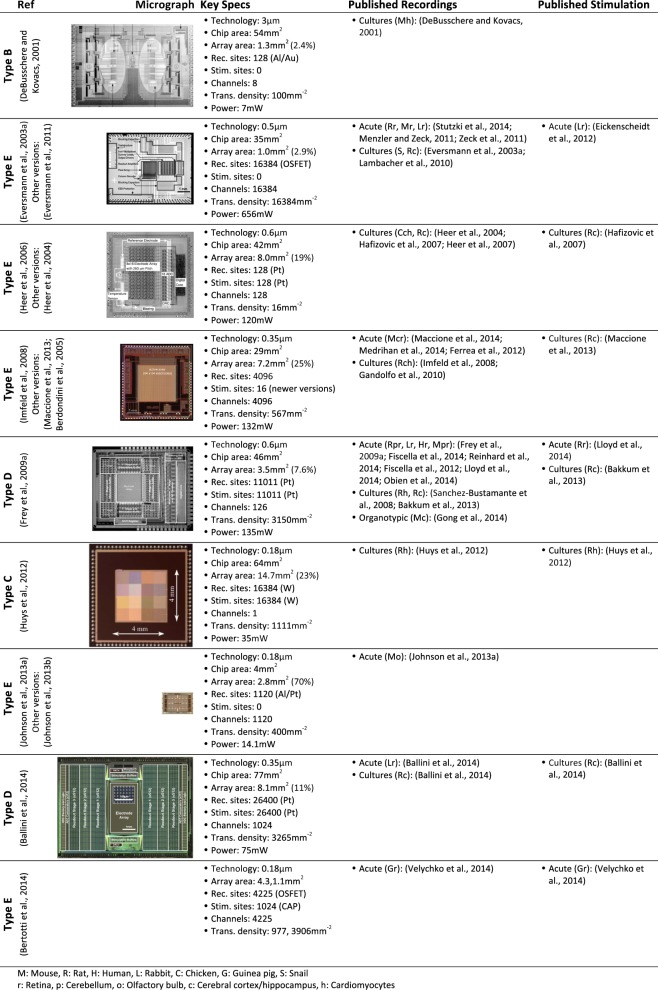
**CMOS-based *in vitro* MEAs**. CMOS-based *in vitro* MEAs, their key specifications and references to biological applications for recording and stimulation are listed in this table. The application list includes only one representative citation for each type of preparation. The specification for each device are taken from the reference listed on top and may differ for other versions of the device.

*In vitro* CMOS MEAs have already been used in a wide variety of applications, for recording, for electrical stimulation or for both. Figure 5 lists *in vitro* CMOS MEAs, their key specifications and preparations for which they have been used so far. Some additional *in vitro* CMOS-based MEAs that are not listed in Figure 5 can be found here: (Tokuda et al., 2006; Greve et al., 2007; Meyburg et al., 2007; Yegin et al., 2009; Johnson et al., 2012). In addition, the functionality of some *in vivo* CMOS MEAs has also been demonstrated using *in vitro* applications (Aziz et al., 2009).

The two most prominent preparations investigated with *in vitro* CMOS MEAs so far are acute retina preparations from mice (Menzler and Zeck, 2011; Fiscella et al., 2012; Maccione et al., 2014), rats (Eickenscheidt et al., 2012; Lloyd et al., 2014; Stutzki et al., 2014), rabbits (Zeck et al., 2011; Ballini et al., 2014; Fiscella et al., 2014), guinea pig (Velychko et al., 2014) and humans (Reinhardt and Blickhan, 2014); and cultured neuronal cells from snails (Eversmann et al., 2003a), rats (Hafizovic et al., 2007; Heer et al., 2007; Gandolfo et al., 2010; Lambacher et al., 2010; Bakkum et al., 2013; Ballini et al., 2014) and chicken (Hafizovic et al., 2007). Additionally, data from acute slices of the cerebellum (Frey et al., 2009a; Obien et al., 2014), cortex (Ferrea et al., 2012; Medrihan et al., 2014) and olfactory bulb (Johnson et al., 2013a) have been shown. Also cultured cardiomyocytes were studied (DeBusschere and Kovacs, 2001; Heer et al., 2004; Imfeld et al., 2008; Sanchez-Bustamante et al., 2008; Huys et al., 2012) and first results from mice organotypic slices were presented (Gong et al., 2014).

Certainly, *in vitro* CMOS-based MEAs, being still an emerging technology with commercial availability only starting recently, have a high potential for future biomedical research and diagnostics (Jones et al., 2011).

## Understanding MEA signals

Here, we describe the parameters that contribute to neuronal signal transduction from the source into digital form.

### What do microelectrodes detect?

A microelectrode can detect the changes in the extracellular field caused by the current flows from all ionic processes across the morphology of the closest neuron and from other nearby cells, not only neurons (Buzsáki et al., 2012; Anastassiou et al., 2013). The effect of the transmembrane currents on the electric field and the detected potential on a microelectrode depend on the magnitude, sign, and the distance from the recording site (Nunez and Srinivasan, 2006), see Section The extracellular space.

An AP is a biophysical event that occurs once the neuron's transmembrane potential reaches a threshold due to stimuli or other inputs (e.g., synapses, gap junctions). On the other hand, we consider a “spike” to be the signal from a putative AP. For extracellular recordings, spikes are commonly identified as voltage signals that exceed a threshold. During an AP, the initial rapid Na^+^ ion influx creates a sink and results in a large negative spike in the EAP. Thereafter, the slow K^+^ efflux produces a source resulting in a small positive spike. In contrast, IAP first shows a positive spike and later a negative volley. EAPs are usually around tens to hundreds of microvolts in amplitude and <2 ms in duration while IAPs are at tens of millivolts and around the same duration as EAPs (Buzsáki et al., 2012). If IAPs can only be detected by direct access inside the neuron, e.g., patch-clamp, EAPs can be identified when electrodes are placed at the vicinity (~100 μm) of the spike origin (Henze et al., 2000; Egert et al., 2002), usually at the perisomatic area, i.e., around the soma or near the axon initial segment.

Aside from measuring single- and multi-unit spiking activity, electrodes also sample LFPs. The LFP is assessed by the signal content in the low-frequency band of the recorded signal (<300 Hz) (Belitski et al., 2008; Buzsáki et al., 2012), while EAPs are analyzed after filtering the LFP out (300–3000 Hz) (Quian Quiroga, 2009). Although the contribution of EAPs to LFP is still unclear, a synchrony of APs from many neurons can participate in the generation of LFPs (Buzsáki et al., 2012). The current opinion is that synchronized synaptic currents in cortical neurons produce LFPs, through the formation of dipoles (Niedermeyer and da Silva, 2005; Nunez and Srinivasan, 2006). We refer the reader to Einevoll et al.'s extensive review on the modeling and analysis of LFPs for further details (Einevoll et al., 2013). The relationship between LFPs and spikes has also been discussed and studied in several works (Khazipov et al., 2004; Belitski et al., 2008; Montemurro et al., 2008; Minlebaev et al., 2011; Kayser et al., 2012; Cingolani, 2014).

### MEA signal flow

**Figure 6:**
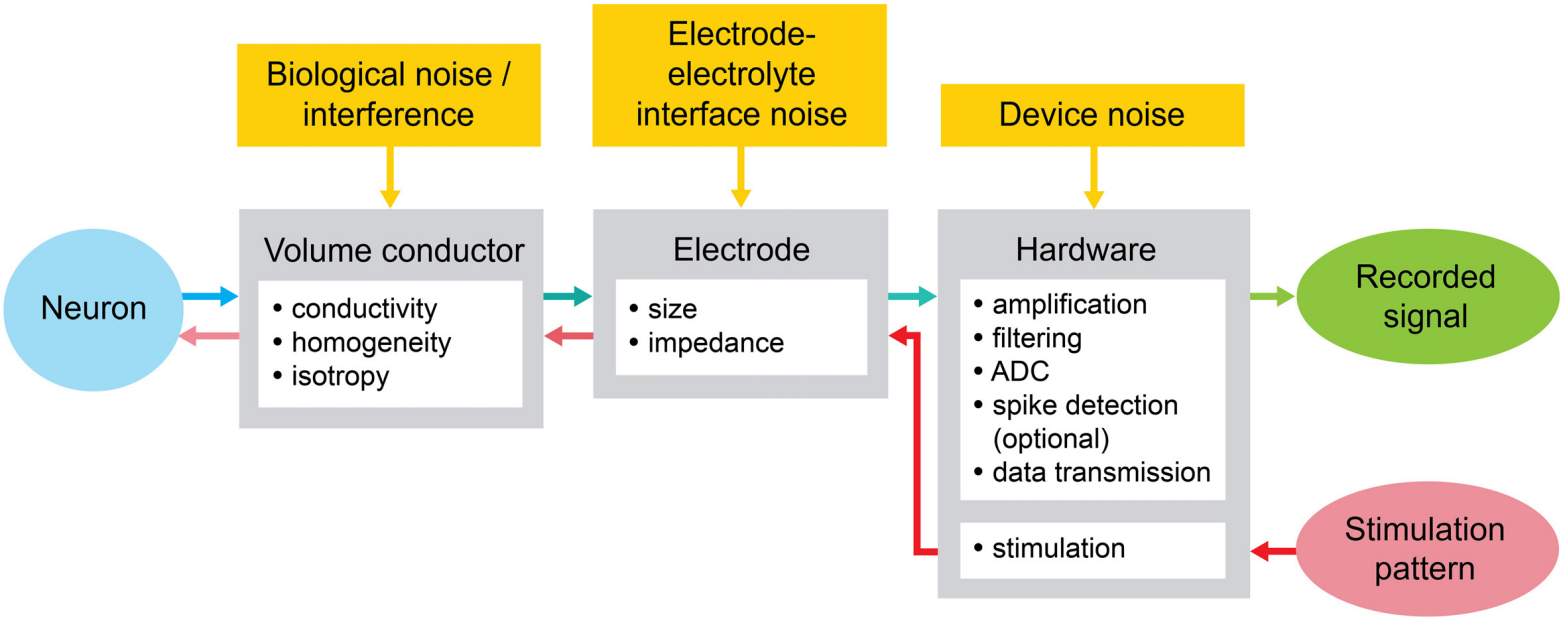
**MEA stimulation and recording system diagram with the noise sources**. The neuron is stimulated by the pulses or waveform generated digitally through the MEA. The response of the neuron, typically an action potential, is transformed by different parameters across the components of the MEA toward the recorded signal.

We consider the components of the MEA recording and stimulation system diagram as shown in Figure 6: (A) the conductive extracellular volume where the electric field caused by neural signal sources forms; (B) the substrate with the embedded microelectrodes; and (C) the hardware connected to the electrodes, including amplifiers, filters, digitizer, data transmission, and stimulator (Stett et al., 2003; Fejtl et al., 2006).

#### Noise and SNR

One crucial aspect of the MEA signal flow is how noise is fed into the amplification chain and how it affects the SNR of the recorded data. SNR is the key specification for the amplifier design, regardless of the actual amplification (Jochum et al., 2009). It is important to consider where the noise, or interference, is injected in the signal chain, as the implications on SNR will differ.

- *Biological noise*. This is a major source of noise stems from the electrical activity of other cells around the recording electrode, e.g., APs of distant cells, but also ionic activity, e.g., subthreshold events in neurites of nearby cells, and synaptic noise due to the stochastic nature of synaptic transmission. Several models of biological noise, or sometimes also called background noise, have been developed by simulating uncorrelated single-unit spiking activities or examining multi-compartmental neuron models located at distances far enough away from the electrodes such that the spikes cannot be resolved (Eaton and Henriquez, 2005; Martinez et al., 2009; Lempka et al., 2011; Jäckel et al., 2012; Camuñas-Mesa and Quian Quiroga, 2013). Although such models replicate the average biological noise in experiments, it is possible that the cell type, size, and morphology, along with the firing rates and correlated activity, can affect the shape of the background signal. For spike analysis, the LFP is also considered biological noise and filtered out.
- *Electrode-electrolyte interface noise*. On top of the biological noise, the liquid-metal interface also adds to noise. At low frequencies, such as below 10 Hz, processes at the electrode generate noise with a steep roll-off of *1/f* or even *1/f*^2^ (Hassibi et al., 2004; Heer, 2005). More relevant for electrophysiology are the frequencies above that, where thermal noise is the main contributor (Gesteland et al., 1959; Liu et al., 2007). The equivalent thermal noise can be calculated as follows:
  $$v_{n}=\sqrt{4\cdot k\cdot T\cdot Re\left(Z_{e}\prime \right)\cdot \Delta f},$$
  where *k* is the Boltzmann constant, *T* is the absolute temperature, *Re*(*Z*′_*e*_) is the real part of the effective electrode impedance (see Section Neuron-electrode interface), and *Δf* is the noise bandwidth. Another source of noise is the 50–60 Hz hum from power lines. This noise is largely picked up between the microelectrode and the connection to the input of the preamplifier, due to its high impedance at that frequency. Hence, minimizing the distance between the electrode and the amplifier is a major design requirement for MEA circuits (Harrison, 2008). Proper grounding and shielding of the MEA setup can minimize interference.
- *Device noise*. Finally, the device or the system that amplifies and digitizes the signals further adds to noise. Usually, the front-end amplifier is the most important factor to consider. A general design objective for such amplifiers is to ensure that the signal acquisition system does not limit the system performance with regard to noise. As discussed above, this is a design tradeoff in which also power and circuit area may play a role. For example, if the maximal allowed contribution to noise from the circuitry is set to 10%, the amplifier noise needs to be 45% or less as compared to the noise of the electrode. A commonly used figure of merit that captures the tradeoff between noise and amplifiers' supply current is the noise efficiency factor (NEF) proposed in Steyaert and Sansen (1987). This figure has also been adapted to capture the different supply voltages used to allow a better comparison with respect to power consumption, coined the power efficiency factor or PEF (Muller et al., 2012). For *in vitro* MEAs, area is also of critical importance, as it usually impacts electrode density and total channel count. The efficient use of the overall area is reflected in the ratio of the actual array area divided by the overall chip area (see Figure 5). Quantization noise is another noise contributor of the hardware. It originates from the discretization error made at the ADC part of the MEA system. As an approximation for the quantization noise, typically a value of $\frac{1}{\sqrt{12}}$ times the magnitude of the least significant bit (LSB) is used. Typical ADCs applied for MEA systems have a minimum of 8-bit resolution, with systems that employ off-chip ADCs often using 16-bit or higher resolution. The transmission of data may also affect the quality of the recorded signal, e.g., if a lossy compression has to be used due to bandwidth constraints.

#### The extracellular space

The analysis of EAPs and LFPs usually assume a homogeneous, resistive extracellular space based on the volume conductor theory, i.e., Kirchhoff's current law or charge conservation and Ohm's law (Nunez and Srinivasan, 2006). The difference in waveforms of a signal recorded at different locations in the tissue is mainly due to how each neuronal source linearly sums up, with source contributions weighted inversely proportional to their distance (Nunez and Srinivasan, 2006). Under the assumption of a purely homogeneous, isotropic, and ohmic extracellular medium, Maxwell's equations of electromagnetism can be rewritten with appropriate Laplace boundary conditions, such that for a single point current source the following equation holds true for the potential at an electrode, *V_e_* (Klee and Rall, 1977; Nunez and Srinivasan, 2006; Anastassiou et al., 2013):

$$V_{e}=\frac{I}{4\pi \sigma r},$$

where *I* is the point current, σ is the conductivity of the medium, and *r* is the distance between the point source and the recording electrode. Since the membrane currents are distributed over the cable-like morphology of a neuron, a line source approximation (LSA) of current sources was also proposed (Holt, 1997; Gold et al., 2006; Einevoll et al., 2007).

The presence of numerous cell bodies, dendritic structures, axonal bundles, blood vessels, and white matter in brain tissue raises questions as to whether the brain can really be considered as purely ohmic. Moreover, the frequency spectra observed in LFP and EEG (Pritchard, 1992; Freeman et al., 2003; Bédard et al., 2006a; Buzsáki, 2006; Bédard and Destexhe, 2009; Miller et al., 2009; Milstein et al., 2009) led to uncertainties regarding the role of extracellular space in frequency dependent filtering. Pettersen and Einevoll (2008) clarified that in a purely resistive and homogeneous extracellular medium, amplitude variability and low-pass filtering of EAPs occur due to the spatial separation of correlated current sources and sinks during a spike. Similarly, Lindén et al. (2010) found that an intrinsic dendritic low-pass filtering affects the LFP, not the extracellular space. Other interesting studies described how low-pass filtering effects can be achieved in a medium of radially decaying conductivity (exponential) around the source (Bédard et al., 2004, 2006b).

Already in 1968, Robinson (1968) suggested that inhomogeneities, such as the presence of glial cells in brain tissue, can considerably impact the extracellular recording of spiking activity. He also argued that since the resistance of the paths around the glial cells are lower (for signals at 1 kHz) than the paths through them (due to the membranes), the extracellular signals would flow between the cells, not through them. Thus, the structures in the tissue can cause directional differences in the conduction of signals (Rice et al., 1993; Okada et al., 1994). Similar results were achieved by Nelson et al. (2013) across fiber and cell obstructions. Various studies explored different properties of brain tissue conduction, such as anisotropy (Nicholson and Freeman, 1975; Logothetis et al., 2007); anisotropy and inhomogeneity (Ranck, 1963a,b; Hoeltzell and Dykes, 1979; Goto et al., 2010); and capacitive property (Gabriel et al., 1996a,b; Bédard et al., 2004; Bédard and Destexhe, 2009). Whole brain analysis of the electrical tissue properties at the microscale may be useful for modeling and analyzing EAPs and LFPs from different groups of neurons in different brain areas. Using the four-point probes method (Kelvin sensing, with separate pairs of current-carrying and voltage-sensing electrodes) is advisable for measuring the electrical impedance of brain tissue, since it minimizes the influence of the impedance of the current carrying electrodes.

#### Neuron-electrode interface

**Figure 7:**
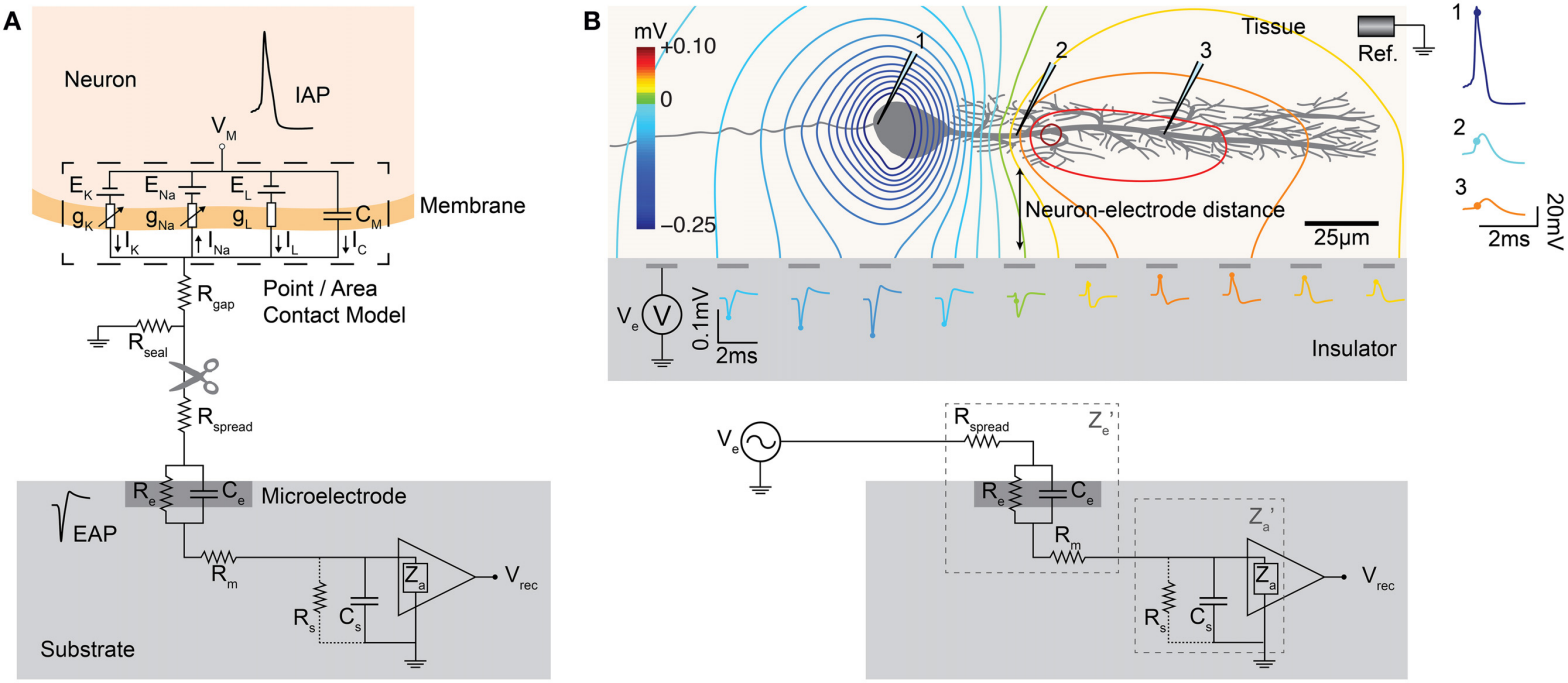
**MEA neuron-electrode interface. (A)** The classic point or area contact model derived from Fromherz (2003). The cell membrane is represented with an equivalent model based on the Hodgkin-Huxley model of the squid axon (Hodgkin and Huxley, 1952). C_M_ represents the capacitance across the neuronal membrane, i.e., the lipid bilayer. The voltage-gated ion channels (K for potassium and Na for sodium) are represented by non-linear conductances, g_K_ and g_Na_, and the leak is shown as a linear conductance, g_L_. The reversal potentials that drive the flow of ions are represented by E_K_, E_Na_, and E_L_. The ion flow is shown by I_K_, I_Na_, I_L_, and I_C_. The other elements are described in the text. V_rec_ is the recorded voltage signal. Typical IAP and EAP recordings are shown. The location of the scissors indicates where the “cut” can be made to separate the neuron-electrode interface into two parts. **(B)** Generalized neuron-electrode interface separating the problem into two parts. Upper—“Fluid”-side: The potential at the electrode sites can be solved using the volume conductor theory. The MEA surface is assumed to be an insulator such that the method of images can be applied on Coulomb's law to solve the potential at any point on the MEA surface. The neuron-electrode distance influences the signal amplitude measured at the electrodes. High spatial resolution allows for recording at several locations of a single neuron, with large negative spikes located at the perisomatic area and positive spikes at the dendritic area, i.e., return current. Lower—“Metal”-side: The voltage measured at the electrode is shaped by the electrical parameters of the electrode-electrolyte interface, represented by *Z*′_*e*_ as the effective electrode impedance and *Z*′_*a*_ as the effective input impedance. This model is derived from Robinson (1968), Nelson et al. (2008), Hierlemann et al. (2011).

Using an equivalent circuit model, the interface between neurons and microelectrodes *in vivo* has been described and characterized by Robinson (1968). Later, this concept has been adapted for substrate integrated MEA devices, e.g., to compare metal microelectrodes with OGFET devices in simulations (Grattarola and Martinoia, 1993). This representation of the neuron-electrode interface was then coined the *point-contact model* (Weis and Fromherz, 1997) and is shown in Figure 7A. It is a standard model of the electrical characteristics of the interface, which has also been extended to an *area-contact model* (Buitenweg et al., 2003; Fromherz, 2003) to consider the spatial distributions that can accurately describe the interface at subcellular resolution. Detailed characterizations of the electrode model for various materials have been carried out, see Section Electrodes and Transducers. Other studies on similar neuron-electrode equivalent circuits were conducted by Ingebrandt et al. (2005), Joye et al. (2008), Thakore et al. (2012). These models assume that a tight seal between the neuron and electrode is needed to measure EAPs from isolated neurons. In the *in vivo* situation, such close contacts usually do not exist and models usually focus less on the electrode properties themselves, but more on the electric field generated by current sources in a conductive volume (Lind et al., 1991; Moffitt and McIntyre, 2005; Gold et al., 2006). For HDMEAs, such volume conductor models match measurements for, e.g., the idealized case of point source in saline (Obien et al., 2013), but also for complex neuronal morphologies in acute brain slices (Frey et al., 2009a). In cell cultures, it has been observed that EAPs are also detected by electrodes that do not have a tight seal with the isolated neuron, even by electrodes that are relatively distant from the neuronal source (Bakkum et al., 2013). Thus, we generalize the neuron-electrode model in Figure 7B, which applies to tissue slices and dissociated cell cultures.

One important assumption for this generalization is that we can treat the MEA surface as an insulator allowing us to separate the neuron-electrode interface problem into two parts: (i) “fluid”-side and (ii) “metal”-side. We are able to do this separation because the high input impedance of MEA amplifiers largely prevents any effect of the metal electrode on the potential at the “fluid”-side of the interface. This is valid, as long as the impedance on the “metal”-side seen by the electrode is much larger as compared to the tissue or fluid impedance at all frequencies of interest. The generalized interface model can then be interpreted such that an electrode detects the average voltage present at the recording site, as claimed by Robinson (1968), Nunez and Srinivasan (2006), Nelson et al. (2008). The detected voltage is then shaped by the electrical characteristics of the interface. It should be noted that the model, as shown here, is adapted for the recording situation, focusing on the understanding of the neuronal signals as recorded by MEAs. Similar models have also been developed and used for the application of electrical stimulation using microelectrodes or capacitive stimulation spots, as discussed in Section Stimulation.

***“Fluid”-side: voltage at the electrode by volume conduction***. For simple geometries of the “fluid”-side, assuming that the MEA surface is an insulating infinite plane and the fluid a homogenous, isotropic medium, we can apply the method of images to the point-source equation given in Section The extracellular space, such that the potential *V_e_* at any given electrode *e* can be solved using the following equation (Obien et al., 2013:

$$V_{e}=\frac{1}{2\pi \sigma }\sum \frac{I_{n}}{r_{n}}.$$

*I_n_* represents the *n^th^* point current source and *r_n_* represents the distance between the point source and the recording electrode, with *n* = 1… N, where N is the number of individual point sources. For electrodes larger than an ideal point electrode, *V_e_* can be solved at multiple locations of the surface area of the microelectrode and then averaged. The larger the electrode area, the larger the averaging effect (Grimnes and Martinsen, 2008). Anisotropy can also be incorporated in this model easily (Nicholson and Freeman, 1975). However, more complex geometries of, e.g., the MEA device (such as *in vivo* neural probes) or an inhomogeneous medium generally require a finite element method to solve for the electric field and the potential at the electrode.

The orientation and distance between the neuronal source and the measuring electrode affect the amplitude and shape of the signals detected, as discussed in Section The extracellular space. The spread and decay of the signal over the MEA surface plane is highly correlated with the distance of the signal source from the surface. This makes it possible to estimate the distance between a current source and the MEA electrodes by measuring the voltages at high spatial resolution using an HDMEA (Obien et al., 2013). The same concept can be applied to estimate the neuron-electrode distance given a good model of the membrane currents of the neuron being recorded (Somogyvári et al., 2005, 2012; Frey et al., 2009b; Delgado Ruz and Schultz, 2014).

***“Metal”-side: signal transformation by the electrode-electrolyte interface***. The “metal”-part of the model is an equivalent circuit of the microelectrode modified from Robinson (1968), Franks et al. (2005), Nelson et al. (2008), Hierlemann et al. (2011). In this model, the input to the circuit is a low impedance voltage source with the value corresponding to the potential resulting from the currents in the volume conductor discussed above. This voltage (*V_e_*) is connected to the effective electrode impedance *Z*′_*e*_, consisting of *R_spread_*, *R_m_*, *R_e_*, *C_e_*. *R_spread_* is the *spreading* resistance, which is the resistance a current sees, that spreads from the microelectrode into the electrolyte. Its value is mostly dependent on the electrode geometry and the electrolyte conductivity. *R_e_* and *C_e_* are the resistance and capacitance, respectively, of a simplified model of the electric double layer that forms at the electrode-electrolyte interface. This is a reduction of the more complex model, consisting of a constant-phase-angle impedance, a charge-transfer resistance, and a Warburg impedance. *R_m_* is an additional resistance representing the metallic part of the microelectrode.

The effective amplifier input impedance, *Z*′_*a*_, is connected in series to *Z*′_*e*_, which includes the actual input impedance of the amplifier *Z_a_* and the shunting paths to ground outside the amplifier (*R_s_* and *C_s_*). Input amplifiers are designed to have a high *Z_a_* (above 10 MΩ at 1 kHz) to limit the influence of *Z_a_* on the measured voltage (Robinson, 1968). The shunt resistance (*R_s_*) is usually negligible, but the shunt capacitance (*C_s_*) reduces *Z*′_*a*_, especially at higher frequencies (Robinson, 1968; Nelson et al., 2008). *C_s_* is the combination of all capacitances from connectors and wires from the bath to the amplifier, and the capacitance from metal of the electrode (through the insulation) to the bath (Robinson, 1968). The ratio of *Z*′_*e*_ (mostly *C_e_*) and *Z*′_*a*_ is of importance, so if the electrode impedance is low enough, the influence of shunt capacitance to the signal is small (Robinson, 1968; Nelson et al., 2008). HDMEAs require small electrodes to achieve a high resolution, and therefore also the *C_e_* is usually small. However, monolithic integration allows keeping *C_s_* small too. For example, *C_s_* is estimated to be below 0.5 pF for the HDMEA presented in Frey et al. (2010), whereas passive MEA can have a significantly larger parasitic capacitance, depending on the thickness of the insulation and the track width [e.g., James et al. measured values of 60–100 pF (James et al., 2004) and Nisch et al. estimated it to be below 15 pF (Nisch et al., 1994)]. For measurements requiring a high accuracy despite having a device with a large *C_s_*, capacitance compensation circuits can be used, as those commonly used in patch-clamp amplifiers and, e.g., also used for highly accurate tissue impedance measurements (Logothetis et al., 2007).

#### Effect of electrode size and density

Sizes of published microelectrodes range from 5 to 50 μm in diameter (Kim et al., 2014). Larger electrodes have a higher possibility of getting physically near the neurons and of picking up higher amplitude spikes (Camuñas-Mesa and Quian Quiroga, 2013), e.g., studies by Moxon (1999), Paik et al. (2003), Ward et al. (2009), Andersen et al. (2010) claim that larger recording electrodes can record from more neurons simultaneously. However, large electrodes (>50 μm diameter) can average out a neuron's spatially localized peak signal amplitude with nearby smaller amplitude signals. This reduces the peak signals, which can result in a lower SNR. Electrode size also affects the electrode impedance *Z*′_*e*_, which in turn determines electrode noise (see Section Noise and SNR). With that, there are three effects for which SNR improves with larger electrodes (reduced electrode noise, reduced attenuation due to large *Z_e_*/*Z_a_* ratio, and increased chance to “being at the right spot”), and one effect for which SNR gets worse with larger electrodes (increased signal averaging).

As discussed above, for EAP recording in the 300–3000 Hz frequency band, electrode noise is mostly thermal and comparably small, especially if some sort of electrode coating is used and the electrode size is >5 μm in diameter. Without considering electrode noise, Camuñas-Mesa et al. studied via simulation the optimal electrode size for an *in vivo* situation, considering neuronal background activity. For their simulation parameters, they found 40 μm to be the optimum (Camuñas-Mesa and Quian Quiroga, 2013). For HDMEAs, the situation is a bit different. Most importantly, there is no need to enlarge the electrode to be close to the location with the largest signal, as there will always be another electrode “at the right spot”. Secondly, the effective input capacitance can be significantly smaller as compared to passive devices, due to a small *C_s_*, which in turn allows for a smaller *C_e_*. As a result, small electrodes are much more preferable in this situation, with only electrode noise being the limiting factor.

LFP and EAP recordings from neurons located distant to the electrodes feature lower spatial frequencies and therefore allow for larger electrodes without signal degradation than recordings from neurons within close proximity. Especially for LFPs, Nelson and Pouget (2010) discussed that the electrode impedance and recording site geometry are not crucial. This is because LFPs only vary in a spatial scale much larger than the size of electrodes used for extracellular recordings, e.g., by a few hundred micrometers (Katzner et al., 2009) or even by 1 mm (Destexhe et al., 1999). In addition, LFPs are of lower temporal frequency, making electrode noise a more important factor as in that range, it is dominated by *1/f*^2^ noise, which makes larger electrodes more favorable.

It is therefore important to choose optimal electrode sizes depending on the targeted application. In addition, a high density of electrodes will inherently limit the electrode size.

## Practical application of microelectrode recordings

Here, we provide a brief overview on how to extract relevant information from distorted, convoluted, and noisy recorded signals. We then review relevant applications of MEAs for the study of single neurons and networks using various techniques and preparations.

### MEA signal processing and spike sorting

MEA signal processing usually includes (1) filtering the raw data traces, (2) spike detection, and (3) spike sorting.

First, the raw signal is processed to separate the fast APs from LFP and noise by applying a band-pass filter (Quian Quiroga, 2007), with a typical narrow band of 300–3000 Hz. Filtering methods aim to attain higher SNR and lower false positive rates. The filtering process can add phase distortions and therefore alter the shape of the detected EAP. One can avoid such phase distortions by using non-causal filters when future inputs are also used for computation. In hardware implementations and online filters, causal filters are typically used though, as non-causal filters would require the usage of a data buffer (Quian Quiroga, 2009). Depending on the scientific goal, good practice is to record data with wide-band filters (e.g., 1–7000 Hz) and negligible phase distortion, then apply the narrower band filters only for the purpose of the extraction of spike timing information, for which undistorted spike shapes are not needed. One can then still use the spike timing information generated by the spike sorter to re-extract the undistorted spike shapes from the original data.

Once the signal is filtered, the spikes are detected. Amplitude thresholding is commonly used, although other spike detection methods have been implemented, e.g., two-point procedure (Borghi et al., 2007; Maccione et al., 2009) and template-matching (Kim and McNames, 2007). The threshold is usually set as a multiple (5 times) of the baseline noise level, calculated as the root mean square (RMS) of the signals with a mean value of zero. In the presence of many spikes, the threshold can be estimated using a measure based on the median, which is less sensitive to outliers and therefore more robust with regard to spike frequency (Quian Quiroga et al., 2004).

After spike detection, spike shapes are grouped according to their spike shape, which is referred to as spike sorting. Several feature extraction techniques have been used, e.g., principal component analysis or PCA (Quian Quiroga, 2007) and wavelet transform (Mallat, 1989). In the ideal case, distinct neurons will have spikes whose features belong to well-separated clusters, and each neuron will only be part of one cluster. In practice, spike sorting often requires user supervision in order to manually evaluate the performance of the procedure and correct for errors, e.g., to merge nearby clusters or remove outliers. For a detailed explanation of the spike sorting steps, the reader is referred to other review articles (Lewicki, 1998; Einevoll et al., 2012a). Available spike sorting packages and frameworks include Wave_Clus (Quian Quiroga et al., 2004), NeuroQuest (Kwon et al., 2012), SigMate (Mahmud et al., 2012), UltraMegaSort (Hill et al., 2011), EToS (Takekawa et al., 2010, 2012), and QSpike tools (Mahmud et al., 2014), among others. HDMEAs can improve spike sorting performance since with high-resolution spatial information, one can more efficiently separate individual neurons (Gray et al., 1995; Jäckel et al., 2011; Franke et al., 2012).

A number of concerns have been raised regarding the effectiveness of spike sorting. In fact, it is difficult to validate spike sorting algorithms and it is important to test them based on realistic simulated data (Einevoll et al., 2012b). For *in vivo* experiments, or in acute recordings where the electrodes can move with respect to the neurons, drift may occur and alter the recorded signal. Another issue is the amplitude variability of APs from a single neuron that can lead to clustering errors, either intrinsically or due to bursts (McCormick et al., 1985; Henze et al., 2000; Delescluse and Pouzat, 2006; Stratton et al., 2012), such that one cluster may contain the large amplitude spikes and the second one the smaller amplitude ones (Van Dijck et al., 2012).

### Using MEAs for neuroscience studies

MEA recordings have been employed to understand neuronal communication, information encoding, propagation, and processing in neuronal cultures as well as in brain slices and retina explants (Taketani and Baudry, 2006). Recent works start to take full advantage of the unique abilities of HDMEAs.

#### Bursts

Bursts and burst rates of APs in a neuron or across a network of neurons is a common feature extracted from data in MEA applications. Bursts have several meanings and functions in neuroscience, e.g., synchronization, information carrier, and motor pattern generation. Single neurons can exhibit *bursting*, or burst firing, when APs fire at a high frequency for a period of time, followed by a quiet period. Bursts can be triggered by the network activity (environment) or can be intrinsic to the neuron (phenotype of the cell). There are many algorithms to detect the presence of bursts from single neurons (see Samengo et al., 2013; Bakkum et al., 2014 for some methods).

Besides single neuron bursting, population-wide synchronous activities are also of interest. For example, repetition of activation patterns (Abeles and Gerstein, 1988; Sun et al., 2010) can be considered as memory traces, replayed by the appearance of a similar stimulus or due to internal processes that occur, e.g., during sleep (O'Neill et al., 2008; Abel et al., 2013). Bakkum et al. (2014) investigated parameters for and compared the performance of various burst detectors on population-wide bursts. An inter-spike interval (ISI) based network burst detector was able to identify small and large bursts better than other techniques in cultured networks. Rate-based detectors detected larger bursts only, while prematurely identifying the end of bursts. See Kreuz (2013) for further details and methods on quantifying synchronization.

#### MEAs and neuronal cultures

**Figure 8:**
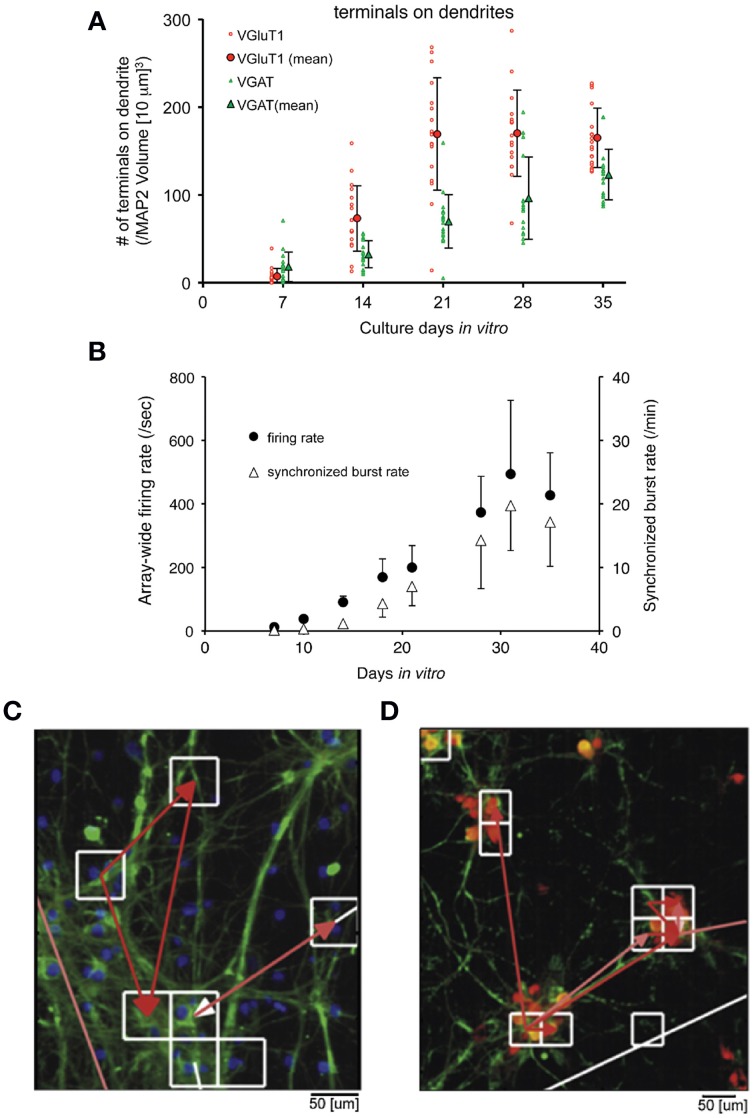
**Neuronal culture studies using MEAs. (A,B)** Combination of MEAs with immunostaining and microscopy to analyze the relationship between the development of synapses and electrical activity of neurons, adapted with permission from Ito et al. (2013). **(A)** Plot showing the number of synapses along the neuronal dendrites in a long-term primary culture. The glutamatergic (red) and GABAergic (green) synapses along the dendrites of neurons were obtained by immunostaining from cultures at 7–35 days in vitro (DIV). The number of synapses at the dendrites continuously increased for 3 weeks and saturated afterwards. The same is true for synapses at the soma (not shown), which saturated after 30 DIV. **(B)** Plotted data from MEA recordings of a long-term culture. A similar pattern is observed from the firing rate and synchronized burst rate measured by a MED64 MEA device from 7 to 35 DIV. Both the firing and burst rates increased until 30 DIV, which eventually saturated afterwards. **(C,D)** Application of HDMEAs to analyze the functional connectivity of neurons *in vitro*, adapted with permission from Maccione et al. (2012). Fluorescent images of stained neurons on an HDMEA are shown with arrows indicating the functional connectivity (from white—weak to red—strong) obtained by analyzing spike trains using cross-correlation.

Since Pine reported the first MEA recordings from dissociated neuronal cultures in 1980 (Pine, 1980), the method has been expanded for pharmacological tests, diagnostics, and investigation of neuronal growth and connectivity. Combination of immunostaining, fluorescence microscopy, and MEA recording allows the identification of neuronal types and synapses, e.g., GABAergic and glutamatergic, and the analysis of neuronal electrical activity in long-term cultures. Using this technique, Ito et al. (2013) observed a correlation between synapse densities and electrical activity of cultured rat cortical networks (Figures 8A,B). The initial increase in glutamatergic and also GABAergic synapses was accompanied with increasing electric activity, which reached a plateau after 28 days in culture when the synapses reached their final density.

More complex neuronal culture analyses can be done using HDMEAs such as burst pattern tracking (Gandolfo et al., 2010) and functional connectivity estimation (Maccione et al., 2012). By plating low-density cultures, it is feasible to not only optically visualize the network of stained neurons, but also to estimate the functional connections and to obtain detailed functional maps at cellular resolution (Maccione et al., 2012), see Figures 8C,D. Maccione et al. processed and analyzed the HDMEA signals by ad hoc developed spatio-temporal filtering and by applying a cross-correlation based method.

#### MEAs and brain slices

**Figure 9:**
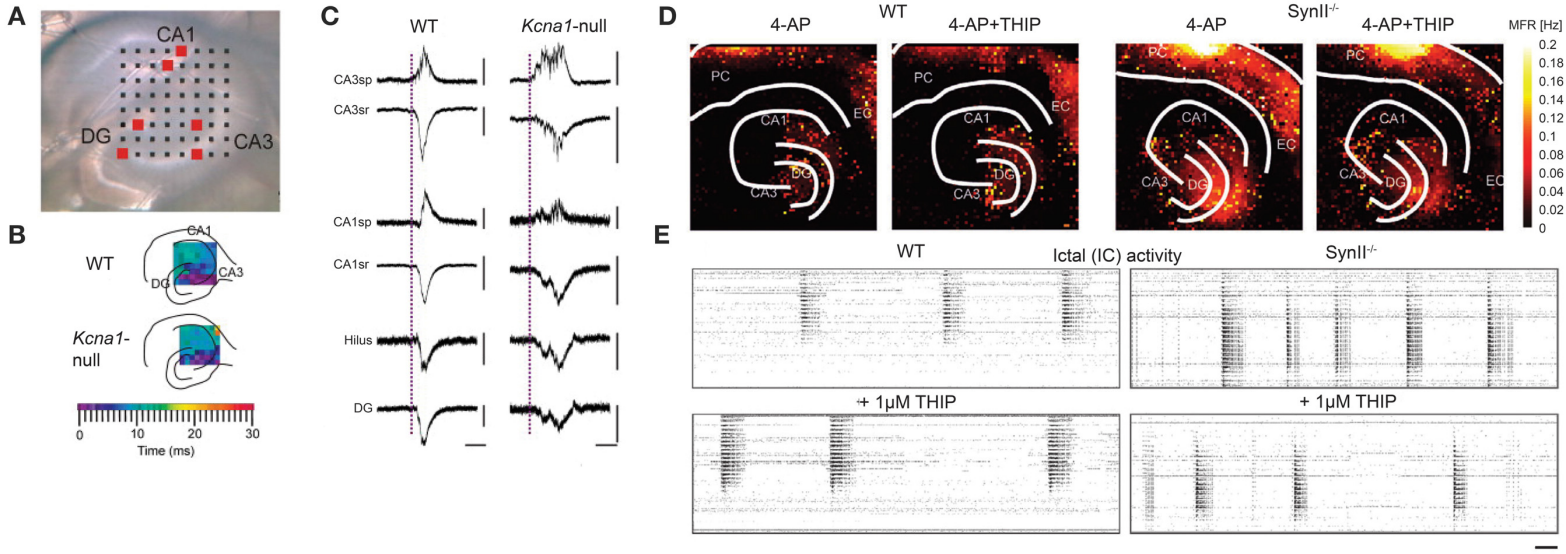
**Waves in acute hippocampal slices revealed by MEAs. (A–C)** Studying the effect of the delayed rectifier potassium channel α-subunit Kv1.1 to sharp waves in *in vitro* hippocampal slices using MEAs, modified with permission from Simeone et al. (2013). **(A)** Image of a *Kcna1*-null (knock-out of the gene encoding Kv1.1) hippocampal slice on an MEA. Black squares correspond to the electrodes. The regions of the hippocampus are also indicated. **(B)** The sharp waves in wild-type (WT) and *Kcna1*-null hippocampi are initiated in CA3 that spread with similar time-courses. **(C)** Representative sharp waves from WT and *Kcna1*-null hippocampi recorded at the location of red boxes in **(A)**. The sharp waves are longer (with ripples) in *Kcna1*-null compared to WT. Scale bars: horizontal, 50 ms; vertical, 50 μV except for WT CA3sp (100 μV), WT CA3sr (200 μV), KO CA1sp (20 μV), and WT CA1sr (200 μV). CA, cornus ammonis; DG, dentate gyrus. **(D,E)** Studying the effect of deleting synapsin II (Syn II) to the tonic inhibition in mouse hippocampal slices using HDMEAs, adapted with permission from Medrihan et al. (2014). **(D)** Mean firing rate computed from each electrode from WT and Syn II knock-out hippocampal slices before and after THIP treatment. THIP: (4,5,6,7-tetrahydroisoxazolo[5,4-c]pyridin-3-ol; gaboxadol), a selective agonist of δ subunit-containing GABAA receptors. **(E)** Raster plots showing highly synchronized bursts, x-axis corresponds to time, y-axis corresponds to pixels (electrode). THIP reduced the high frequency bursts in Syn II knock-out hippocampus. Scale bar: 1 min.

A brain slice is a 3D environment of neurons that can be placed on MEAs to monitor electrical activity. Cutting the brain into very thin slices has allowed access to neurons deep in the brain for imaging, i.e., mapping the anatomy. The same method can be used for recording the activity of neurons that are otherwise difficult to reach and identify *in vivo*. This requires a setup to keep the neurons viable, i.e., by perfusion with artificial cerebrospinal fluid (ACSF) with continuous carbogen (95% oxygen and 5% carbon dioxide) gassing. The neurons and network structure in slices are physiologically and biochemically more similar to the *in vivo* situation. It is possible to observe LFPs and oscillations inherent in different states of the brain. Such recordings have been done for different brain areas, e.g., hippocampus, suprachiasmatic nucleus, etc. For instance, MEAs have been employed to investigate the disruption of normal network waves and oscillations in the brain caused by the absence of certain ion channels in neurons. In one particular case, Simeone et al. studied the effect of the delayed rectifier potassium channel α-subunit Kv1.1 to the oscillations in the hippocampus shown in Figures 9A–C (Simeone et al., 2013). By reducing or eliminating the expression of Kv1.1 in the axons of the hippocampal tri-synaptic pathway, the authors were able to observe an increase in occurrence of fast ripples (80–200 Hz bandwidth, 50% longer duration) and high frequency oscillations associated with epilepsy, as shown in Figure 9C. Similar applications have been done using HDMEAs. Medrihan et al. (2014) showed that the absence of synapsin II (Syn II), a protein related to epilepsy, decreases tonic inhibition in mouse hippocampal slices, thus increasing synchronized bursts (see Figures 9D,E). THIP (4,5,6,7-tetrahydroisoxazolo[5,4-c]pyridin-3-ol; gaboxadol), a selective agonist of δ subunit-containing GABAA receptors, restores tonic inhibition.

**Figure 10:**
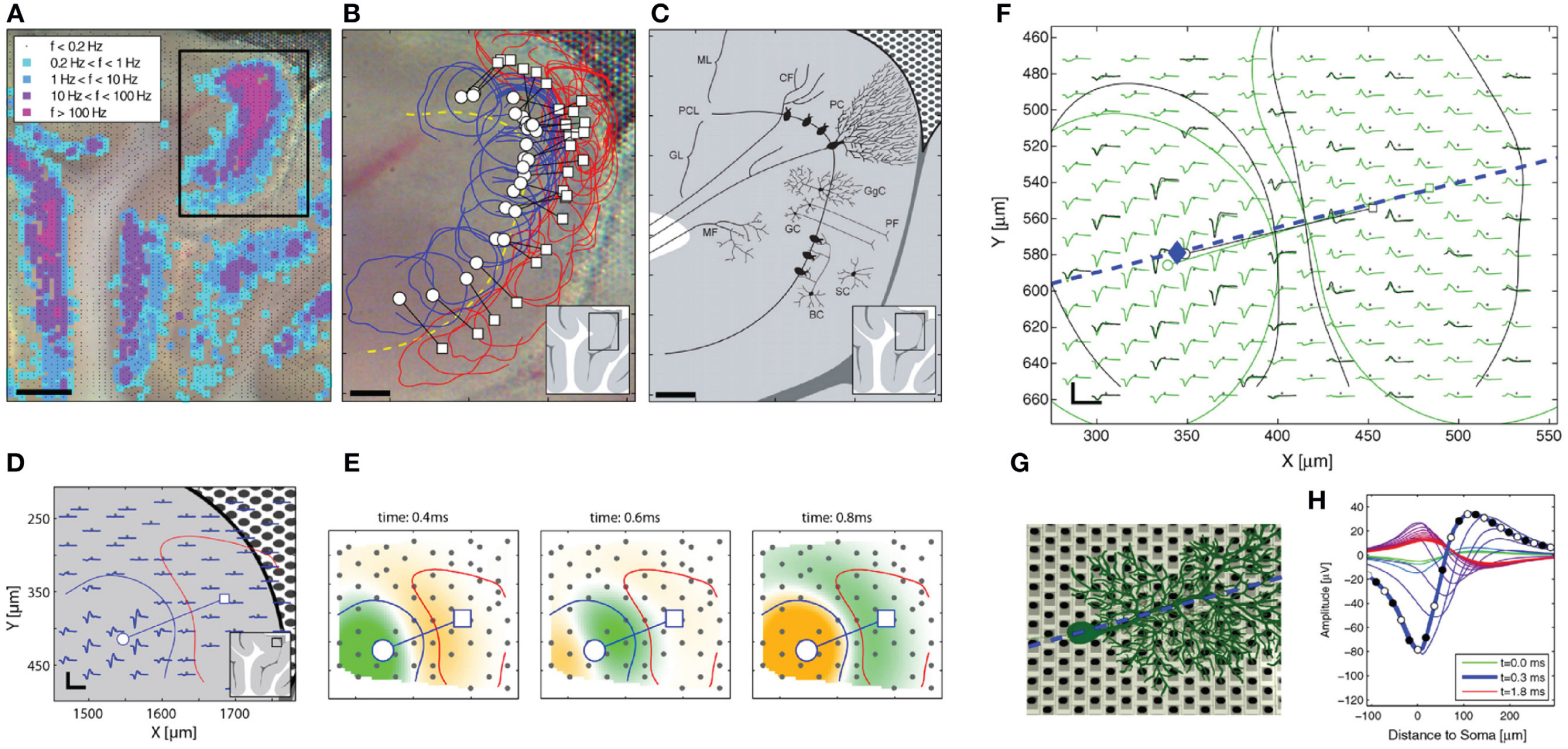
**High-resolution mapping of spontaneous Purkinje cell activity using HDMEAs. (A–E)** HDMEA recordings from an acute slice preparation of the caudal half of the cerebellar vermis. **(A)** Activity map of the detectable spike activity in the recording area. Small dots correspond to the electrodes used for recording (~30% of the available electrodes). Events exceeding a threshold of ±36 μV were used to calculate the color-coded event rate. Scale bar: 0.3 mm. **(B)** Close-up of a region with high activity delimited in **(A)**. All units identified by spike sorting are marked, i.e., the somatic region is blue and the dendritic region is red. Scale bar: 0.1 mm. **(C)** Schematic of the basic cellular structures in the cerebellar slice (Gray, 1918). Scale bar: 0.1 mm. ML, molecular layer; PCL, Purkinje cell layer; GL, granular layer; CF, climbing fiber; MF, mossy fiber; PF, parallel fiber; PC, Purkinje cell; GgC, Golgi cell; SC, stellate cell; BC, basket cell. **(D)** Footprint of a PC selected from the region shown in **(B)**. Scale bar: vertical is 200 μV, horizontal is 1.9 ms. **(E)** Current source density (CSD) analysis for the cell shown in **(D)** at several points in time (green: sink; yellow: source). The sink moves from the soma at 0.4 ms to the proximal dendrites at 0.6 ms and covers the dendritic area, while the soma repolarizes. Frequency band: 180 Hz–3.5 kHz. **(F–H)** Matching simulated and measured EAP footprints. **(F)** Comparison of the recorded average single-unit spikes (black traces) and the spikes calculated from a compartment-model simulation of a PC (green traces). Scale bar: vertical is 100 μV, horizontal is 1.9 ms. **(G)** Illustration of the position and orientation of the simulated PC, with the center of the soma located [blue diamond in **(F)**] 40 μm above the chip surface. **(H)** Simulated potential on the chip surface along a line parallel to the soma-dendrite axis [dashed blue line in **(F,G)**] during the spike evolution at 0.1 ms intervals. The black and white dots on the potential line of maximal amplitude (bold blue line) represent the HDMEA spatial resolution (18 μm pitch). Significant spatial undersampling of the potential distribution curve can be observed by reducing the lateral spatial resolution by 50% (black dots only, pitch 36 μm), especially for the largest negative peak. All panels and descriptions adapted with permission from Frey et al. (2009a).

Depth recording of EAPs from neurons up to 100 μm distance from the MEA surface was also shown (Egert et al., 2002; Frey et al., 2009b). Subcellular resolution recording from single Purkinje cells (PCs) in acute cerebellar slices was demonstrated using HDMEAs (Frey et al., 2009a). One important factor is to ensure tissue adhesion on the MEA surface. Adhesion can be achieved by cellulose nitrate coating (Egert et al., 2002), but also by a slice anchor typically used for patch-clamp recordings. EAPs were observed along the PC layer and, after spike sorting, the EAP footprint of a single PC was analyzed. The negative spikes were recorded around the perisomatic area of the neuron, while positive spikes were obtained along the molecular layer corresponding to the dendrites of the PC. A comparison of the high spatiotemporal resolution recording with simulations of a full-compartmental model based on the stereotypical morphology of a PC was done. Figure 10 shows both measured and simulated EAP data from PCs at high resolution. Although the planar geometry of PC is advantageous, similar results might be obtained from neurons in other brain areas.

Aside from acute preparations, MEAs have been used to analyze the brain function using organotypic slice cultures. For example, Ito et al. studied the functional connectivity in hippocampal and cortical organotypic cultures (Ito et al., 2014). They analyzed the network activity at different frequency ranges using the wavelet transform of the cross-correlogram.

#### MEAs and retina

**Figure 11:**
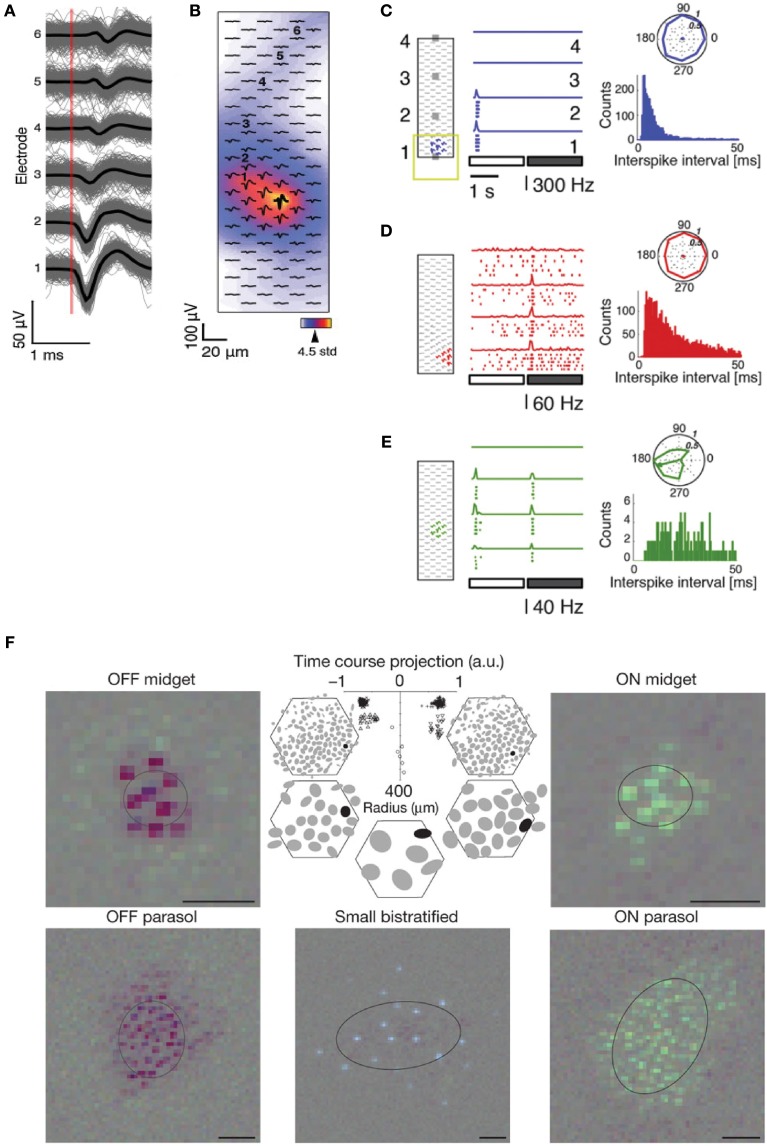
**Identification of retinal ganglion cell receptive fields using HDMEAs. (A–E)** Characterization and analysis of HDMEA recordings from defined populations of mouse retinal ganglion cells (RGCs), adapted with permission from Fiscella et al. (2012). **(A)** Each trace shows the average (thick black lines) of the 959 superimposed EAPs (gray lines). The electrode locations are indicated in **(B)**. The propagation speed of the spike was calculated to be 0.7 m/s. **(B)** Footprint of an RGC over an area of 0.025 mm^2^. The highest peak-to-peak amplitude is shown by the thick dark waveform. **(C–E)** Physiological response of RGCs. Left panel: RGC footprint on a recording block of the HDMEA. The yellow square indicates the location of the light stimulus, with the gray squares indicating the center of the stimulus at four positions. Middle panel: Raster plots corresponding to four stimulation locations indicated in the left panel. Each dot corresponds to a single EAP. Each raster plot shows the response to five repetitions of the same stimulus. The firing rate of the RGC (averaged from five responses) is indicated below. Right panel top: Polar plot showing the responses of the RGC to motion of a bar in 8 directions at 45° radial intervals. Right panel bottom: Inter-spike interval distribution showing the time intervals between consecutive spikes. **(C)** Blue = ON RGC. **(D)** Red = OFF RGC. **(E)** Green = ON-OFF RGC. **(F)** Classification of RGC types and receptive fields at single cone resolution, adapted with permission from Field et al. (2010). The RGCs were recorded simultaneously and classified using the responses to white noise stimuli. Top middle panel: Receptive field radius vs. the first principal component of the response time course. The clusters reveal different RGC types. Surrounding panels: Identified RGC types highlighted at the top middle panel. The RGCs are stimulated with fine-grained white noise to reveal single cone receptive fields. Scale bars: 50 μm.

The planar arrangement of retinal ganglion cell (RGC) bodies and axons is highly compatible with MEA recordings from retina explants. Responses of RGCs can be recorded using different types of light stimulations (Segev et al., 2004; Wässle, 2004; Jones et al., 2011). This allowed the identification of cell types of populations of RGCs and the mapping of their receptive fields (Meister et al., 1994; Chichilnisky, 2001), in different regions of the retina. Fiscella et al. (2012) established a methodology applied to mice retina that uses light stimulation and HDMEAs to identify, select, and record from defined populations of RGCs. After spike sorting the HDMEA recordings, the EAP footprints of detected RGCs were obtained, as shown in Figures 11A,B. Each detected RGC is assigned to one of the four types of ON–OFF direction-selective RGCs, depending on the occurrence of the response to different light stimulation patterns (see Figures 11C–E).

Another study on retina (macaque) using HDMEAs revealed the identification of the type, location, and strength of the functional input of each cone photoreceptor to each RGC (Field et al., 2010). Populations of midget, parasol, and small bistratified RGCs were recorded simultaneously in the presence of white noise “visual” stimulation. The spatial receptive field and response time of RGCs were detected by computing the spike-triggered average of the stimuli. Afterwards, the detected clusters of cells obtained by PCA were further stimulated with 10-fold smaller pixels (5 × 5 μm^2^) to reveal finer details of the receptive fields. The method was able to map putative cones accumulated across the receptive field of RGCs, which were verified by overlaying a microscopy image of cones labeled with peanut agglutinin (see Figure 11F). The authors were able to quantify the strength of connectivity between different RGC types and different types of cones (sensitive to red, green, or blue). These exhibit the capability of HDMEAs, combined with advanced stimulation and analysis techniques, to resolve the functional connectivity of neurons in the retina at single-cell resolution. There are also other recent works on population coding in the retina using MEA recordings (Marre et al., 2012; Tkačik et al., 2014).

#### MEAs and axonal signal tracking

**Figure 12:**
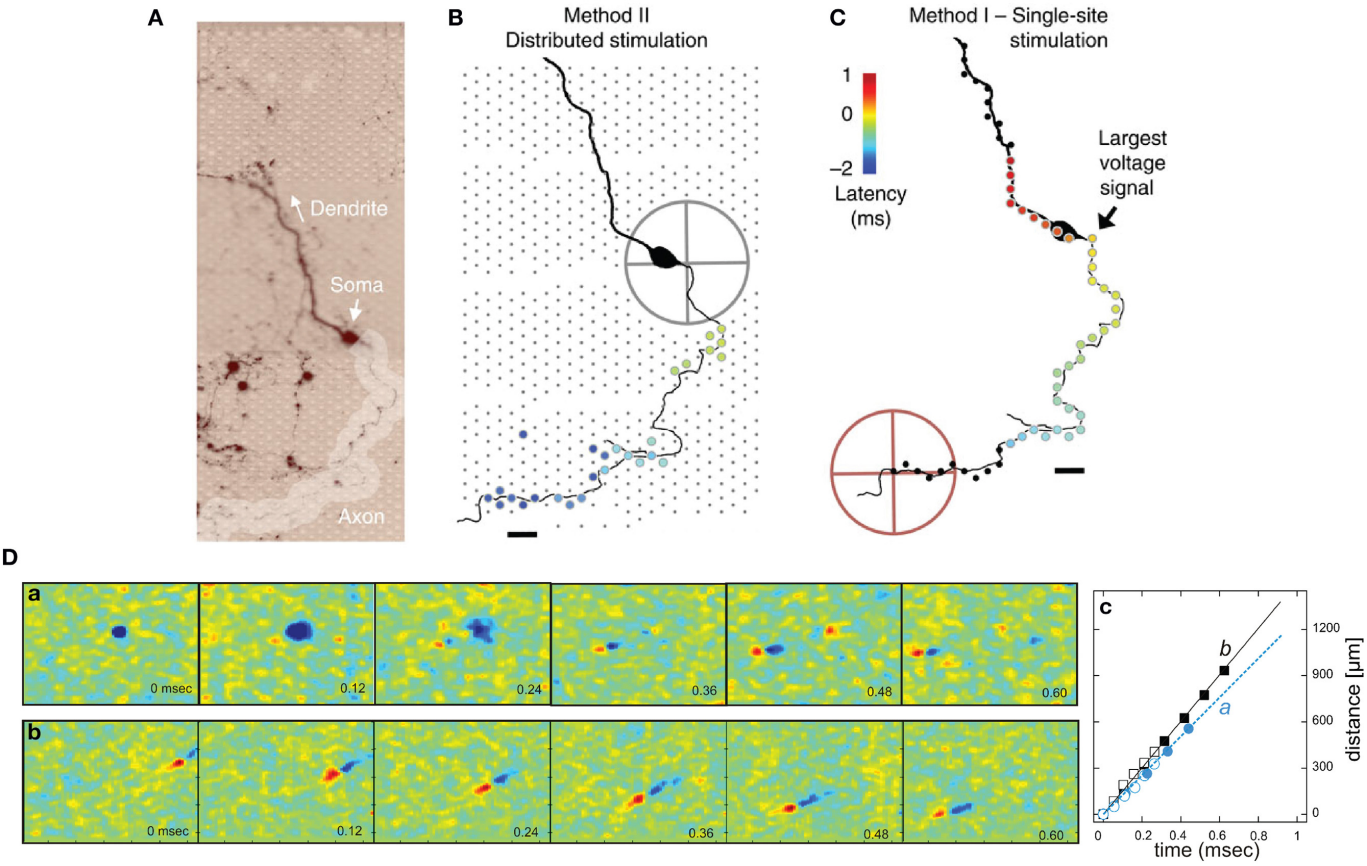
**Imaging axonal signal propagation using HDMEAs. (A–C)** Axonal propagation of a cultured neuron on an HDMEA, adapted with permission from Bakkum et al. (2013). **(A)** Live image of a neuron at 21 DIV transfected with red fluorescent protein (RFP). The axon is highlighted. **(B)** Illustration of the distributed stimulation method. The crosshair represents the location of the “somatic” AP observed while stimulating different electrodes represented by colored dots (color represent the median latency until AP detection, where light gray corresponds to electrodes that did not evoke an AP). The small dots represent the location of the HDMEA electrodes. Scale bar, 40 μm. **(C)** Illustration of the single-site stimulation method. The red crosshair represents the stimulated electrode. The colored dots represent the latencies of detected APs with respect to the largest voltage signal indicated by the arrow. Scale bar, 40 μm. **(D)** Axonal propagation of an RGC from rabbit retina, adapted with permission from Zeck et al. (2011). Consecutive electrical images of the EAP propagation allow for the calculation of axonal conduction velocity. **(a)** Image of a somatic AP (blue spot in the first window) propagating along the proximal axon. **(b)** Image of a biphasic spike recorded from an axon. **(c)** Plot indicating the distance traveled of the AP in time. Open symbols represent data calculated from recordings at 16.4 kHz; closed symbols are recordings at 8.2 kHz.

Taking advantage of the spatiotemporal resolution and high signal quality of HDMEAs, tracking the propagation of APs between cells can be performed. Bakkum et al. (2013) achieved this in dissociated neuronal cultures (see Figures 12A–C). Axonal signals are difficult to identify using conventional methods: thin axons are difficult to patch and extracellular signal amplitudes are rather low compared to those from the soma. A major accomplishment of this work is the capability to electrically image the propagation of APs along axons, across the topology of the whole neuronal network. By using HDMEAs that can record and dynamically stimulate at defined locations, with little artifact to the signals, it was possible to quantify the direction, velocity, and extent of axonal AP propagation. The stimulation and recording techniques are shown in Figures 12B,C. This is a suitable platform to study the role of axons in neuronal computation in the future.

Axonal conduction was also measured by Zeck et al. (2011) from rabbit retina using HDMEAs. The authors were able to measure the velocity of axonal AP caused by stimuli and discovered that similar RGC types respond with the same latency and conduct with similar velocity (see Figure 12D). Except for the area where axons are myelinated, axonal signals were detected from all stimulated RGCs. This work also shows that when axons are very near or flat on the electrode array surface, it is possible to map the flow of APs. The axons do not necessarily need a tight contact on the electrodes, since the potential due to the APs was also detected from other surrounding electrodes, with lower amplitude compared to the electrode nearest the axon.

### Neuronal modeling and HDMEA recordings

Computational modeling is useful to interpret the dynamics and processing of neurons and networks. MEA recordings are commonly analyzed to model neuronal networks (Taketani and Baudry, 2006; Kreuz, 2013; Samengo et al., 2013). Here, we focus on the use of HDMEA data to analyze and model single neurons.

#### Localization of neurons

Neuronal circuits are arranged with high spatial precision and specificity and therefore, spatial information is an important factor in deciphering neuronal activity. Microscopy, fluorescent markers, and transgenic animals have enabled researchers to localize and classify neurons in a high-throughput manner. Together with dynamic multineuron Ca-imaging using spinning-disk confocal microscopy with two-photon excitation, spatial and functional information can be obtained simultaneously. However, the temporal resolution of MEA recordings can capture neuronal responses better than these imaging technologies (Delgado Ruz and Schultz, 2014) and the optical tools described above may not be applicable to all experiments, e.g., due to the unavailability of the transgenic animals, the duration of the experiment, optical access such as in *in vivo* experiments with freely moving animals, etc. Therefore, localization of neurons in MEA recordings has been of interest for *in vivo* and acute slice *in vitro* experiments too.

Based on the volume conductor theory several current source density (CSD) methods have been proposed to solve for the current sources and sinks from LFP and EAP data (Nicholson and Freeman, 1975; Mitzdorf, 1985; Plenz and Aertsen, 1993; Okada et al., 1994; Pettersen et al., 2010; Łęski et al., 2011). A volume CSD approach for measurements using a 3D MEA has also been done (Riera et al., 2014). These methods approximate the location of the sources prior to solving the CSD and may not be suitable for localizing single neurons. Different methods to localize single neurons depend on the source models used, e.g., monopole source type models such as exponential decay and inverse power law models (Blanche et al., 2005; Chelaru and Jog, 2005; Kubo et al., 2008), dipole models (Blanche et al., 2005; Mechler and Victor, 2012), line source models (Somogyvári et al., 2005, 2012), and simplified line model fitted to the perisomatic area of a full-compartmental neuron model (Delgado Ruz and Schultz, 2014).

**Figure 13:**
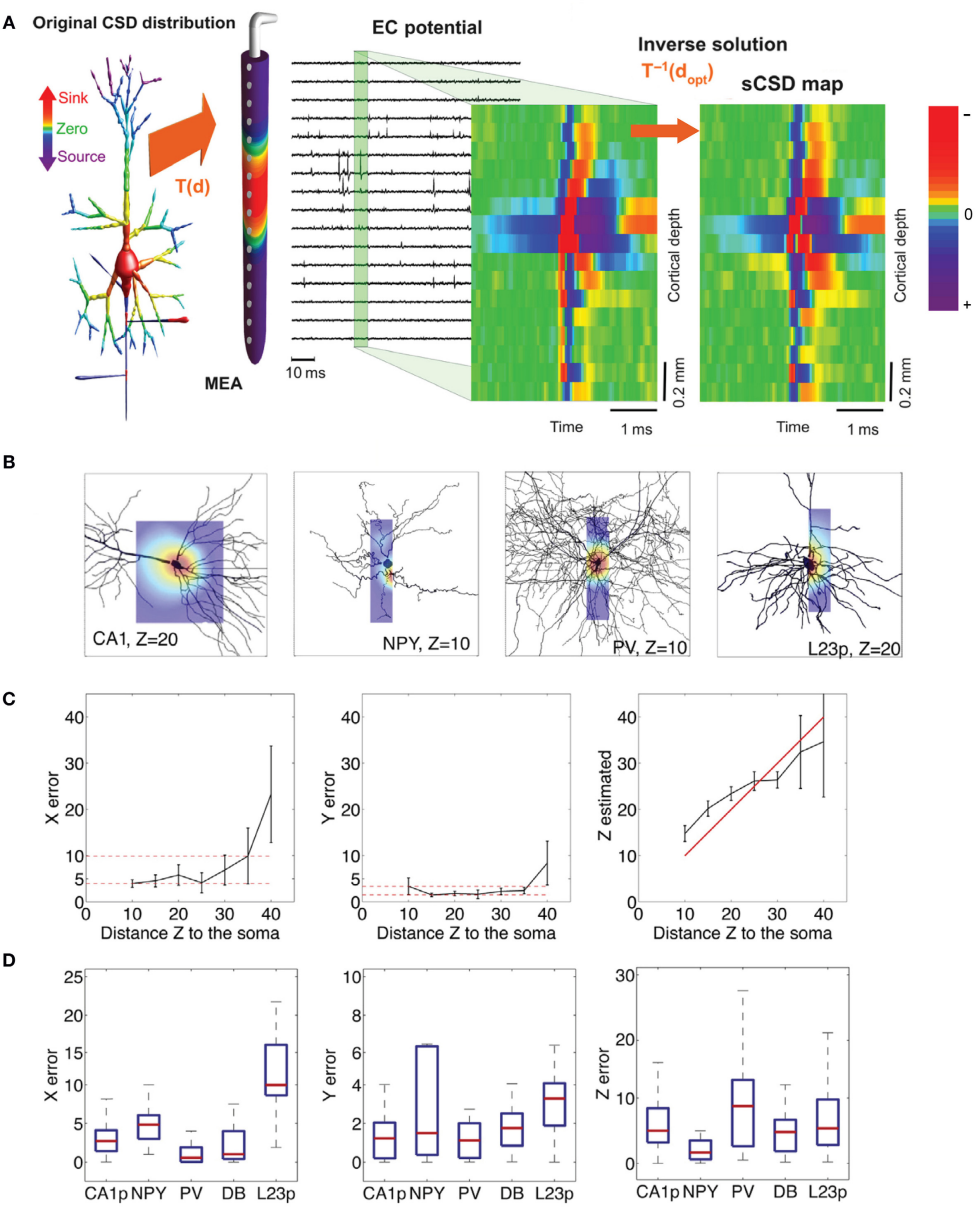
**Localization of single neurons. (A)** Spike current source density (sCSD) method by Somogyvári et al. (2012), figure modified with permission. The experimental setup is shown on the left, where the neuron is oriented at a distance *d* parallel to the *in vivo* MEA. The highest amplitude comes from the current sources at the soma of the neuron (sink) and is detected by multiple electrodes. The forward solution at *d* is given by the T(*d*) matrix, which transforms the CSD on the neuron to the EAP detected by the MEA. The EAPs are shown in the voltage traces per electrode, where one spike is plotted as a color map, indicating the spatial EAP pattern in time. The sCSD obtained from the EAP signals by inverse solution T^−1^(*d_opt_*) is shown on the right. The EAP spatio-temporal map is transformed into a series of normalized CSD distributions [*I*(*d*)] with different *d*-values. Localization is done by solving for *d_opt_*. The optimum *d* (*d_opt_*) is chosen as the value where *I*(*d*) is the most spike-like, i.e., similar to the normalized amplitude of the EAP during the whole duration of the spike. Thus, the EAP and sCSD color maps are similar. **(B–D)** Localization of simulated neurons using simplified line model by Delgado Ruz and Schultz (2014), figures adapted with permission. **(B)** The simulated neurons are CA1 pyramidal, L2/3 pyramidal, double bouquet or DB (not shown), NPY interneurons, and PV interneuron. Localization depends on the location of the sodium trough, which corresponds to the moment when currents are concentrated near the soma. As shown by the color map embedded on the neuron morphologies, the sodium trough (red) is displaced from the soma for NPY due to the contribution of the dendritic arbor and axon, leading to higher localization error along the Y axis shown in **(D)**. **(C)** Localization results for CA1, where the errors along X–Z axes remained low for neuron-electrode distances under 35 μm and increased thereafter, especially along the Z axis. **(D)** The localization errors were not similar for all simulated neurons. The differences in morphology and electrophysiology cause the errors, although the maximum EAP (location of sodium trough) is more or less confined to the perisomatic area.

Somogyvári et al. (2012) proposed spike CSD (sCSD) to estimate the CSD after optimizing for the best locations of the sources from the recording electrodes that recreates the spike data (see Figure 13A). The method has been used to analyze recordings from a 16-electrode probe *in vivo*. Although sCSD has been used to solve for the CSD at the optimized locations of the sources, it assumes that the number of electrodes is equal to the number of sources to solve for. The over-simplification of the number of current sources in sCSD results in errors, especially when the orientation of the neuron being analyzed is at an angle with respect to the measuring electrodes.

On the other hand, Delgado Ruz and Schultz (2014) introduced a neuronal-based model for localization, utilizing known current distributions and morphological traits. The method was tested in simulations and *in vivo* recordings using high-density probes. The authors showed that different morphologies and ion channel distributions of neurons elicit different localization accuracies (see Figures 13B–D). This method, however, assumes that the experimenter knows the type (morphology and current distributions) of neurons being measured for localization and that the dynamics of neurons of the same type are stereotypical.

#### Constraining compartmental models

**Figure 14:**
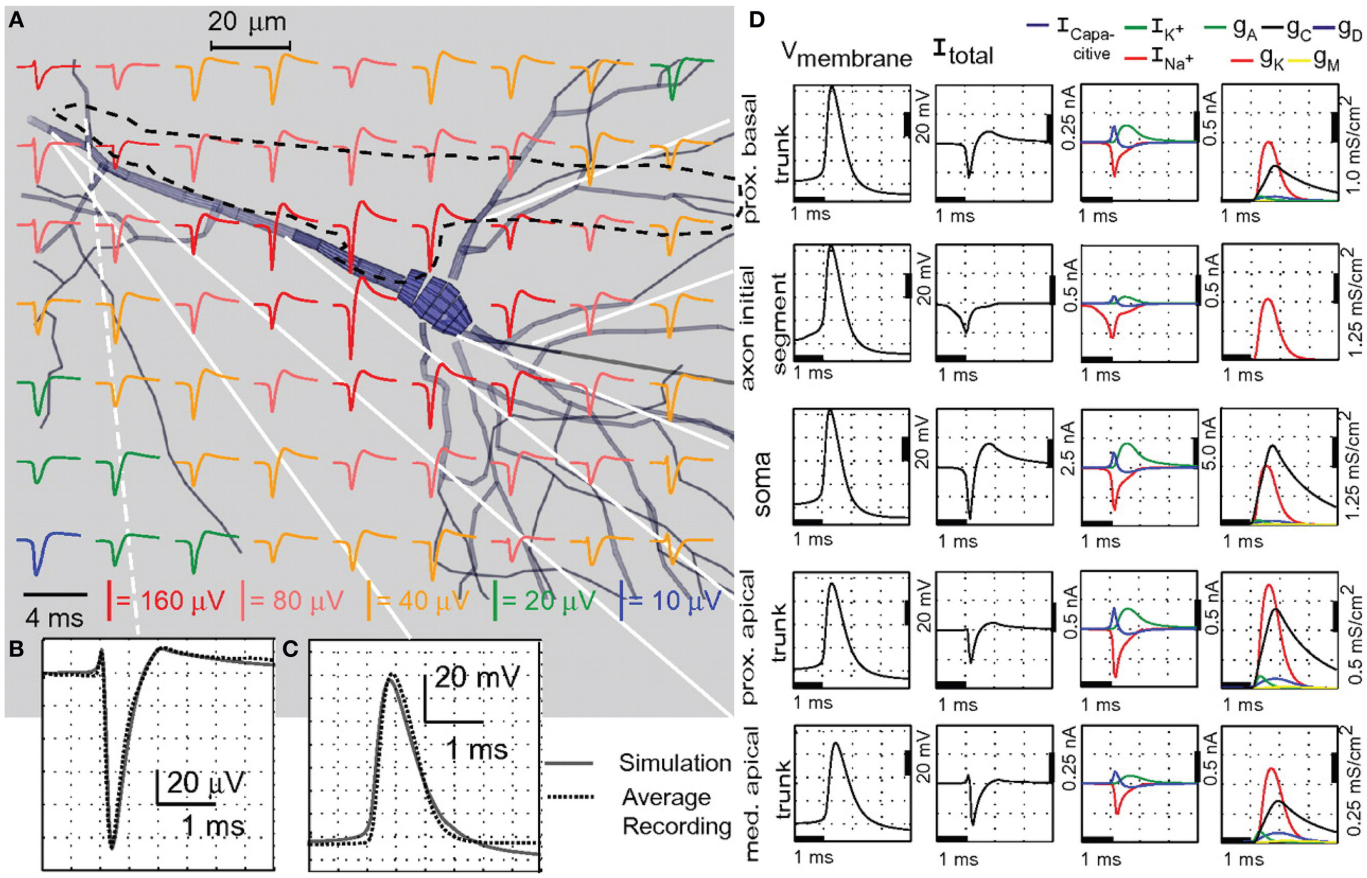
**Ion channel density estimation**. Adapted from Gold et al. (2006). **(A)** The extracellular action potentials (EAPs) solved in a grid from the multicompartmental model of a CA1 pyramidal neuron. The dotted black line indicates the tip of the electrode used to measure the EAPs. **(B)** Enlarged image of the EAP at the electrode tip. Location is indicated by the white dotted line in **(A)**. Solid line in the plot corresponds to the simulated EAP, which is superimposed with the recorded EAP shown as dotted line. **(C)** Comparison of the simulated intracellular signal (solid line) at the proximal apical trunk to the intracellular recording (dotted line). **(D)** First column: The details of the intracellular signal simulation for each compartment. White solid lines in **(A)** indicate the locations of the compartments. Second column: The simulated membrane currents in the same compartments as the first column. The net membrane current across the soma and proximal dendrites best estimates the EAP waveform. Third column: Membrane current components in terms of Na^+^, K^+^, and mixed-ion capacitive current. Last column: Conductivity densities of the A, C, D, K, and M type K^+^ currents. For further details, see Gold et al. (2006).

Aside from localization of neurons, it has also been demonstrated that with known morphology, it is possible to estimate the ion channel density from extracellular recordings. Gold et al. (2006, 2007) simulated realistic extracellular signals based on adjusting the ion channel distributions in full-compartmental models (see Figure 14). With such a method, the EAP waveforms across the neuron's morphology, measured by multielectrodes, can then be used to constrain compartmental models (Gold et al., 2007). Frey et al. (2009a) used this approach to model a full-compartmental Purkinje neuron using HDMEA recordings, see Figure 10. This shows that using high-density EAP recordings, it is possible to model the ion channel dynamics during neuronal function.

## Outlook

We have shown the current status of MEA research in terms of technology, the understanding of signal transduction, and the application to neuroscience studies. After years of MEA development, what is next? One path is to continuously improve the devices, i.e., better SNR, higher spatial resolution, more parallel readouts, scalability, portability, and increased ease-of-use. Additionally, device flexibility and biocompatibility are targets for long-term *in vivo* recording and stimulation. Another approach is to enhance MEA signal pre-processing for experimenters to easily extract meaningful information from recordings in real time. This is crucial for applications where fast, online analysis is required, e.g., closed-loop experiments and brain machine interfaces (BMIs) combined with stimulation therapies.

A promising route is the combination of MEAs with other modalities. Aside from electrical recording and stimulation, brain activity mapping and manipulation at cellular resolution have also been done using optical methods, e.g., fluorescent calcium indicators, genetic markers, optogenetics, two-photon microscopy, etc. Similar to extracellular recordings, the presence of many molecules and compartments in the brain with different optical properties render optical recording and analysis challenging. It is of interest to pinpoint the advantages and constraints of both electrophysiological and optical methods to determine how they can complement each other. Another example is the use of optogenetics to manipulate the activity of specific cellular subpopulations. By using MEAs to measure the response of the cortical circuit at multiple locations during optogenetic manipulation, it is possible to study the functional roles of different classes of neurons (El Hady et al., 2013). Simultaneous multi-scale recording of neuronal electrical activity is also of interest, e.g., concurrent ECoG, *in vivo* MEA, and multiple patch-clamp recordings allow for investigating the relationship between oscillations, LFPs, EAPs, IAPs, and subthreshold activity during different brain states. Additionally, other technologies that can enhance MEA experiments are microfluidics for controlled delivery of drugs, chemical sensing to study the biochemistry involved in neuronal function, and measurement of metabolic processes.

The complexity of the data obtained from all the above mentioned advanced measurement schemes necessitates the application of systems biology techniques for analysis (Ghosh et al., 2011). Computational methods such as multi-scale modeling can combine recordings from different modalities at different time and/or spatial scales into a topological model of a system, e.g., cortical circuit. Through multi-scale modeling, the overall neuronal network activity can be understood, while also having the ability to zoom in to single neurons and even in a specific part of a neuron to study the details of the biochemical and electrical reactions involved. Some works have already started in this direction (Mattioni and Le Novère, 2013). There are already available platforms and packages to develop full compartment models of neurons and neuronal networks based on electrical activity, e.g., NEURON (Hines and Carnevale, 1997) and GENESIS (Bower and Beeman, 1998). There are also tools for modeling biochemical processes, e.g., E-CELL3 (Takahashi et al., 2004), STEPS (Wils and De Schutter, 2009; Hepburn et al., 2012), COPASI (Hoops et al., 2006), SBMLOdeSolver (Machné et al., 2006). The main challenge is to efficiently combine the modules by synchronizing the events properly at different time scales, by matching the spatial information into a topology or morphology, and by using optimization methods to computationally handle such massive amounts of data.

### Conflict of interest statement

The authors declare that the research was conducted in the absence of any commercial or financial relationships that could be construed as a potential conflict of interest.

## Footnotes

^1^Alpha MED Science Co., Ltd. MED64: A low-noise and user-friendly multielectrode array system for *in-vitro* electrophysiology. Available at: http://www.med64.com [Accessed December 1, 2014].

^2^Multi Channel Systems GmbH. MCS: Innovations in Electrophysiology. Available at: http://www.multichannelsystems.com [Accessed December 1, 2014].

^3^The area is calculated as the rectangle of the maximum vertical extend times the maximum horizontal extend, whereas for probes, the horizontal extend is taken as the shaft width.

^4^Only a single sub array of 16 electrodes is considered.

^5^Features a frame rate significantly lower as compared to the other devices.

^6^Noise values are taken from Lambacher et al. (2010).

^7^The authors state that with a new acquisition board, the parallel channel count could be increased to 1024 at 9300 fps.
